# Comparison of SERS Substrates in Direct Analysis of Urine Samples for Detection of Albuminuria: Relatively Large Gold Nano-Spheroids Outperform Gold Nanorods on Gold Film

**DOI:** 10.3390/ijms27198833

**Published:** 2026-10-03

**Authors:** Aigerim Boranova, Almat Kabyl, Bagdat Tuleuov, Madiyar Kassymaly, Zhalaliddin Makhammajanov, Abduzhappar Gaipov, Rostislav Bukasov

**Affiliations:** 1 Department of Chemistry, School of Sciences and Humanities (SSH), Nazarbayev University, Astana 010000, Kazakhstan; aigerim.boranova@nu.edu.kz (A.B.); almat.kabyl@alumni.nu.edu.kz (A.K.); bagdat.tuleuov@alumni.nu.edu.kz (B.T.); madiyar.kassymaly@nu.edu.kz (M.K.); 2 Department of Medicine, Nazarbayev University School of Medicine, Astana 010000, Kazakhstan; zhalaliddin.makhammajanov@nu.edu.kz (Z.M.); abduzhappar.gaipov@nu.edu.kz (A.G.)

**Keywords:** SERS, Raman spectroscopy, nanorods, hot spots, albuminuria, proteinuria, chronic kidney disease (CKD)

## Abstract

Proteins found in human urine can be used as a source of information on kidney function and as possible biomarkers for diseases such as chronic kidney disease (CKD). Focusing on differentiating between high and low albumin concentrations in urine samples, this study examines the roles of various substrates in detecting albuminuria as a specific form of proteinuria using surface-enhanced Raman spectroscopy (SERS). Using gold nanorods (AuNRs) and gold spheroidal nanoparticles (AuNPs) on various substrates to optimize the identification of albuminuria, we studied urine samples from 77 and 124 patients with albumin concentrations above and below 0.3 g/L. SERS spectra were analyzed using principal component analysis—linear discriminant analysis (PCA-LDA), partial least squares with linear discriminant analysis (PLS-LDA), and support vector machine (PLS-SVM)—to differentiate between high- and low-urinary-albumin groups. PLS-LDA was the best-performing method among the three methods. The largest of all tested nanoparticles, 200 nm diameter (D200 nm) AuNPs on gold substrates, performed best in distinguishing between high and low concentrations of albumin in urine, with a maximum average area under the curve (AUC) of 0.97, 100% sensitivity, 92% specificity, and 96% accuracy and a higher enhancement factor of 5.4 × 10^7^ in comparison to the same parameters obtained with λ650 nm and λ750 nm AuNRs on gold, which demonstrated average AUC values of 0.86 and 0.77 and EFs of 11 and 3 × 10^6^, respectively. These larger NPs create hot spots in nanogaps between nanoparticles, which may have better accessibility to protein molecules compared to hot spots created in gaps between smaller nanorods and nanoparticles. This study highlights the role of nanoparticle size and shape in determining optimal SERS substrate parameters, thereby yielding more accurate results in the detection of high concentrations of albumin in urine by direct SERS.

## 1. Introduction

Human urine consists of a large volume of proteins and peptides. In 2021, Swensen et al. identified approximately 2200 proteins and 28,000 peptides in human urine using a liquid chromatography–tandem mass-spectrometry method [1]. Urine proteome analysis involves the comprehensive study of proteins and biochemical processes in urine, analyzed by protein identification methods [2]. Urine is easy to collect in abundant amounts, frequently via noninvasive techniques, for medical proteomics studies. Even though the protein levels in urine are lower than those in other human bodily fluids, including plasma, urine protein composition is comparatively steady and stable [3]. Since urine protein concentrations contain renal system proteins and blood proteins that are excreted from other body systems, protein could be a potential biomarker of kidney diseases [3,4,5]. Chronic kidney disease (CKD) is a worldwide health problem and one of the primary causes of death [6]. In 2017, approximately 843.6 million people were affected by kidney disease, and CKD takes twelfth place on the list of causes of mortality. There are some predictions indicating that the disorder could become the fifth major global underlying pathological cause by 2040 [7]. The assessment of urinary protein biomarkers enables early detection and risk categorization of CKD stages, which may be applied or at least may contribute to the prediction of some other serious health threats such as cancer [8,9]. The identification of early-stage CKD allows for timely therapeutic intervention and facilitates observation of disease progression and mitigation of secondary health complications [10]. Thus, for CKD diagnosis and cancer biological marker identification, the surface-enhanced Raman spectroscopy technique can be used due to its cost-effectiveness, high sensitivity, high sample-processing efficiency, non-labeled detection, and capacity for rapid sample preparation [11,12,13].

Surface-enhanced Raman spectroscopy (SERS) is an enhanced vibrational spectroscopy technique that enables high-resolution structural analysis of small quantities of molecules and the amplification of electromagnetic energy fields occurring due to surface plasmon excitation [14]. SERS is a technique with a high capacity for multiplexing due to its relative narrowness, in comparison to fluorescence peak width, and typically high sensitivity, which can be achieved with relatively simple sample preparation and a non-complicated procedure [14,15]. Enhancement methods for SERS have been studied, and now two mechanisms are known: electromagnetic enhancement via surface plasmon resonance, and chemical enhancement by light-activated charge transfer and interaction between nanoscale surfaces and analytes [16]. SERS can enhance the Raman signal up to 10^14^ and can detect signals even from a single molecule [17,18]. Noble metals show good resistance to corrosion and could amplify SERS enhancement capacity [17,19]. In general, the surface-enhanced Raman spectroscopy technique already shows great potential for widespread implementation in standard clinical analyses, including analysis of urine, since it is a highly sensitive and relatively cost-effective method [4]. As proven by several studies, urine SERS spectra have been used in cancer identification [20,21,22,23]. For example, one study reported 97% sensitivity, 94% specificity, and 96% accuracy in detecting the spectral properties of urine from patients with end-stage kidney diseases using the SERS method, principal component analysis (PCA), and discriminant analysis of principal components (DAPC) [24]. Zong et al. differentiated CKD patients and healthy controls via SERS, principal component analysis, and a linear discriminant analysis algorithm, achieving 78% sensitivity and 86% specificity [25].

The principal component analysis–linear discriminant analysis algorithm is a classical method of spectral data analysis [26]. Commonly, this algorithm is a fundamental technique for data preprocessing, which reduces noise, and classifies and quantifies compounds according to their spectral characteristics [26,27]. Feng et al. utilized the PCA-LDA algorithm to detect nasopharyngeal cancer based on blood plasma SERS [28]. Elsewhere, 99% of coffee samples were accurately assigned to their producers using PCA-LDA analysis based on Proton Nuclear Magnetic Resonance (^1^H NMR) spectra by Charlton’s group [29]. Chen et al. accurately detected prostate cancer by employing the PCA-LDA, whereas Nicolae Leopold’s research group achieved an overall accuracy of 94% in detecting prostate cancer using SERS combined with PCA–LDA diagnostic algorithms [30,31]. Recently, Guo et al. used the PCA-LDA model for the detection and discrimination of dominant apple spoilage fungi and achieved a discrimination accuracy of 98.3% [32]. Aitekenov et al. applied the SERS method for the identification of protein concentrations in human urine using commercial 60 nm and 100 nm gold nanoparticles on four different substrates. The principal component analysis and linear discriminant analysis (PCA-LDA) algorithm and receiver operating characteristic (ROC) analysis yielded a 91% sensitivity, an 84% specificity, 88% accuracy, and an area under the curve (AUC) of 0.94 for the set with 100 nm gold nanoparticles on a Ag substrate. The research outcomes display the technique’s effectiveness in classifying healthy controls and patients with proteinuria for clinical analysis [33].

Partial least squares (PLS) regression is another well-suited algorithm for high-dimensional data where variables outnumber samples [34]. Marigheto proposed a PLS-LDA framework, where partial least squares is first used for feature reduction, followed by two-group linear discriminant analysis for classification [35]. The method demonstrated superior performance compared to a range of conventional classification approaches [36,37]. It resolves multicollinearity by converting predictors into latent variables, and its most common classification extension is partial least squares discriminant analysis (PLS-LDA). For example, Xia et al. employed a PLS-LDA model to analyze serum SERS data, enabling accurate classification of patients with benign and malignant thyroid nodules [38]. In another study, serum SERS was used for the identification of age-related neuropsychiatric disorders, including late-life depression (LDD) and mild cognitive impairment (MCI). Using PLS-LDA, researchers achieved an overall classification accuracy of 83.2%, with higher accuracy for distinguishing healthy individuals from patients (91.6%) and for separating LDD from MCI (85.7%) [39]. Partial least squares-support vector machine (PLS-SVM) is another hybrid classification approach. It combines the dimensionality reduction capability of PLS with the classification performance of support vector machines (SVMs) to identify optimal decision boundaries in complex, high-dimensional feature spaces [40,41]. For instance, PLS-SVM was employed to distinguish oral squamous cell carcinoma (OSCC) tissues from non-oral ones in the study by Sharma et al. [42]. In another study, Zhang et al. obtained diagnostic accuracies of 96.4% and 95.4% with SVM classifier models in discriminating between the serum SERS spectra of bladder cancer patients and healthy controls as well as between low-grade and high-grade bladder cancer patients [43].

Gold nanorods can provide greater SERS enhancement effects in comparison with gold nanospheres in cases where the excitation light is predominantly polarized along the direction of the long axis of the nanorods [44]. The maximum SERS enhancement factor (EF) is achieved when the localized surface plasmon resonance (LSPR) wavelength (λ_max_) is located between the excitation wavelength and the Raman wavelength [45,46,47]. Excitation with a 785 nm laser should drive the EF of nanorods that have a plasmon λ_max_ (650 nm or 750 nm) closer to 785 nm than nanospheres (diameter: 100 nm) with λ_max_ = 569 nm in suspension. AuNRs have both longitudinal and transverse plasmon resonance peaks [48,49]. Because the LSPR peak of the rods is affected by its aspect ratio, modification of the geometric shapes of AuNRs could impact the optical properties in the near-infrared region (700–850 nm). A high aspect ratio shifts a peak to the near-infrared region when near-infrared lasers are used to enhance the Raman signal and avoid fluorescence background [50,51]. For example, Khlebtsov et al. showed simulated surface-averaged EF as a function of the surface resonance wavelengths of AuNRs for a 785 nm excitation laser wavelength. Nanorods with absorption peaks ranging from 700 to 840 nm (with aspect ratios ranging from 2.64 to 3.63) were observed. The rods with an absorption peak at 750 nm showed a 50% improvement in enhancement from 10^4^ to 1.5 × 10^4^, which matched the 785 nm excitation light wavelength [52]. Smitha et al. studied the SERS enhancement factor variation of Au nanorods with different aspect ratios (from 2.4 to 16) using a laser with excitation wavelengths of 514, 785, and 1064 nm. It was found that both the incident laser line and the nanorods’ aspect ratio affect the SERS enhancement factor. Au nanorods with an excitation laser wavelength of 785 nm and an aspect ratio of 16 showed a maximum enhancement of 2.9 × 10^5^, which is an order-of-magnitude increase from 7.69 × 10^3^ [53].

Increasing the size of nanoparticles from an average diameter of 100 nm to an average diameter of 200 nm may bring some significant advantages. Bukasov et al. calculated extinction efficiency (EE) Q_ext_ for nanocrescents with different particle sizes. The EE of 356 nm nanocrescents was greater than that of 194 nm nanocrescents. High EE is an important parameter for sensing, and it is often proportional to the signal-to-noise ratio [54]. Also, according to Transmission Electron Microscopy (TEM) images, nanoparticles may resemble dodecahedrons and have multiple faces and edges where additional hot spots of the electromagnetic field can be formed [19,55,56]. It was demonstrated that an SERS substrate made of particles with edges and irregular surfaces can achieve a higher SERS enhancement factor and therefore may have higher sensitivity in detection than a substrate made of spherical nanoparticles [55,57]. It is known that increasing the size of nanoparticles leads to an increase in electromagnetic field enhancement, resulting in a stronger and more stable electromagnetic field [54,58,59]. For instance, the simulation of E/E_0_ for nanocrescents conducted by Nordlander’s group demonstrates that the larger maximum E/E_0_ (380 vs. 170) was found for nanocrescents that were about two times larger (with inner diameters of 410 vs. 200 nm, respectively) [60]. In general, the SERS signal or SERS EF is proportional to the fourth power of electromagnetic field enhancement [61]. Therefore, x2 larger nanoparticles with an about x2 higher maximum E/Eo are likely to deliver significantly higher SERS enhancement. In fact, Hong and Li observed experimentally that the enhancement factor increases as the size of the gold NPs increases, with the enhancement factor increasing from 2 × 10^5^ for 40 nm AuNPs to 9 × 10^5^ for 80 nm AuNPs [62].

In our research, we compared the SERS spectra of patient urine with relatively high concentrations of urinary albumin with the spectra of patient samples with relatively low concentrations of urinary albumin by analyzing them using PCA-LDA, PLS-LDA and PLS-SVM to calculate sensitivity, specificity, accuracy, and other parameters. We investigated commercial gold nanorod (AuNR) solution, commercial gold, and silver metal substrates for albuminuria identification. Overall, we explored the capacity for improving albuminuria quantification with nanorods and with 200 nm diameter nanoparticles on gold and on other substrates such as silicon, silver film and Al film. There are multiple reports that SERS applications of aluminum foil as a substrate were competitive with applications on gold film [63,64,65]. There are also some reports about sensitive SERS detection on silicon as a substrate [66,67]. Therefore, it is rational to test Al foil and silicon as well as a “classical” SERS-related metal, silver, as more cost-effective alternatives to gold-film-coated substates. This kind of comparative SERS assay evaluating two different nanoparticle morphologies and several different substrates for clinical application of SERS is quite rare among reported applications of SERS.

## 2. Results and Discussion

The objective of this research was to classify individuals with high and low levels of albumin concentrations into groups using received SERS spectral signals and conduct data analysis via PCA-LDA, PLS-LDA and PLS-SVM procedures. Examples of the raw Raman spectra and spectra after correction for a fluorescent background are shown in Figures S1–S3 in the Supplementary Information.

**Figure 1:**
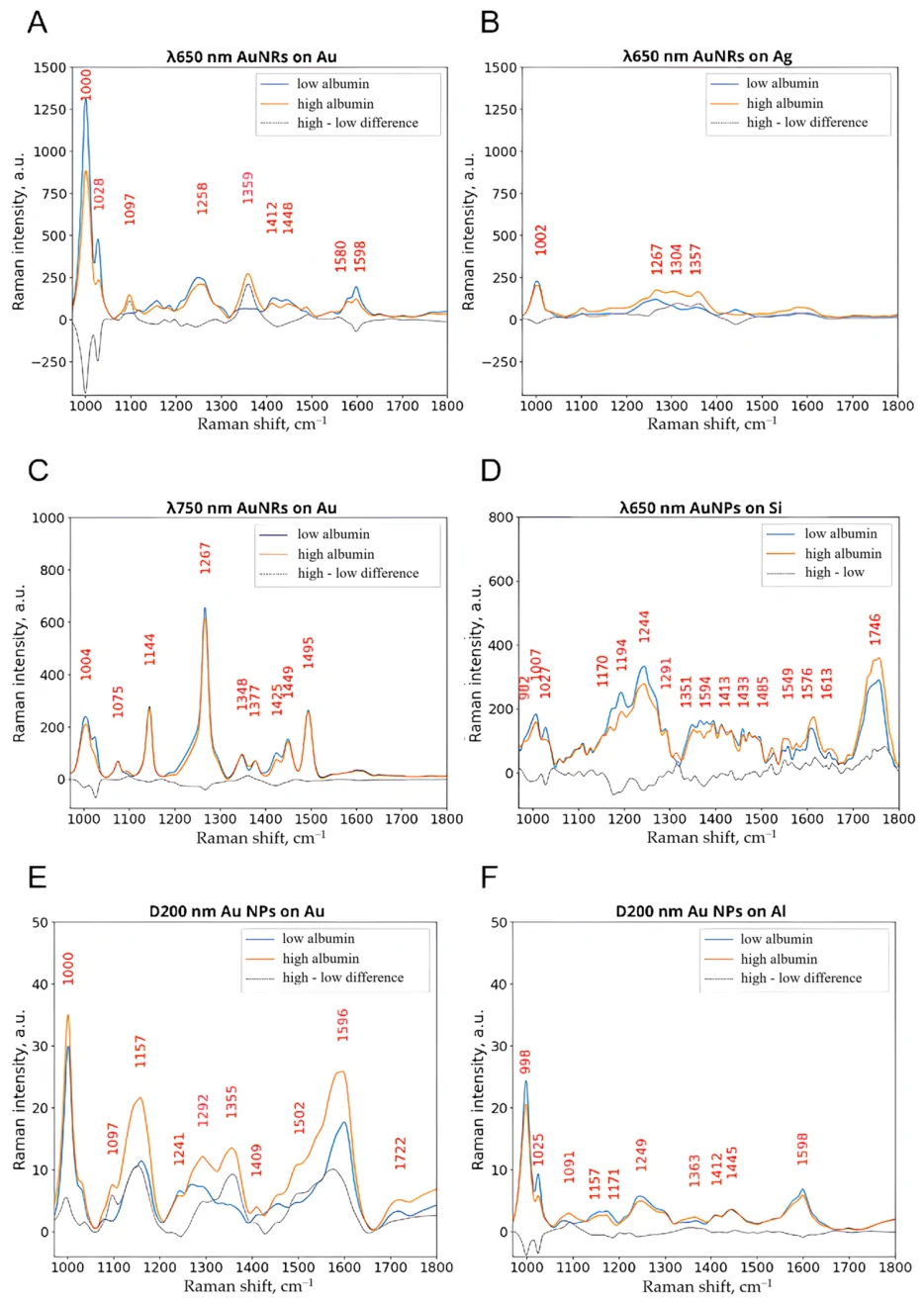
Average SERS spectra of human urine specimens for the high- and low-albumin groups, as well as the spectral representation of their differences for all sets. The albumin concentration threshold (low/high) is 0.3 g/L. (**A**) λ650 nm gold nanorods on the gold substrate; (**B**) λ650 nm gold nanorods on the silver substrate; (**C**) λ750 nm gold nanorods on the gold substrate; (**D**) λ650 nm gold nanorods on the silicon substrate; (**E**) 200 nm diameter (D200 nm) AuNPs on Au; and (**F**) 200 nm diameter (D200 nm) AuNPs on Al.

In Figure 1, there are three average urine SERS spectra for each type of substrate. These include the average SERS spectra of urine with a high albumin concentration, the average SERS spectra of urine with a low albumin concentration, and the difference between the average high and average low albumin spectra (representative average rows of spectra for the high- and low-albumin-concentration groups of patients are shown in the Supplementary Information). The average and normalized Raman spectra represent the urine specimens of 124 individuals. For nanoparticles with a diameter of 200 nm (D200 nm), 77 samples were tested. The cutoff value for differentiation is elevated, and the normal albumin concentration in urine is 0.3 g/L of albumin since, in medical diagnostics, the threshold for regularly used urine reagent strip devices is set at 0.3 g/L [68].

When applying SERS substrates prepared with nanorods, we categorized 64 patients’ samples as having high albumin concentrations, and 60 samples were classified as having low albumin concentrations. For D200 nm nanoparticles, 35 human samples were determined to have high albumin concentrations, and 42 human samples were categorized as having low albumin concentrations.

In Table 1, we characterize the band assignments in the acquired Raman spectra. Peak assignments of the SERS spectra were analyzed based on published studies [69,70,71]. The identified peaks in the spectra are indicative of their composition, which includes substances such as tryptophan, creatine, creatinine, urea, porphyrin, and other compounds.

The spectra, obtained on the gold nanorods, which have surface plasmon resonance (SPR) peaks of λ650 nm and λ750 nm, on gold film and the spectra obtained on the D200 nm gold nanoparticles on gold film are quite similar. This similarity is illustrated by the strongest absorption peak in the range of 1000–1004 cm^−1^. The peaks correspond to the N-C-N stretching in urea. Also, peaks in the range of 1560–1600 cm^−1^ correspond to free and hydrogen-bonded C=O groups in urea [72]. The peaks at 1241–1267 cm–1 correspond to Amide III, which was reported for anti-mouse immunoglobulin G (IgG) and the IgG–anti-IgG complex at 1239 cm^−1^ in the measurement of SERS spectra in the protein antigens used in the experimental study. The peak related to tryptophan is at 1359–1363 cm^−1^ [73]. The peaks at 1177 cm^−1^ and 1348 cm^−1^ correspond to glucose and water, respectively. It can be observed that certain Raman peaks differ from the SERS bands. For example, the SERS peak that we attribute to creatinine at 1409 cm^−1^ is a bit different from the Raman peak at 1397 cm^−1^. Also, the SERS peak observed at 1722 cm^−1^ differs from the Raman peak of amino acids by 19 cm^−1^. It is common for the wavenumbers of SERS and Raman peaks to differ by 10–15 cm^−1^ and sometimes even more. Halvorson et al. noted that peak positions can shift when an analyte forms a bond with the metal [74]. The relative intensities of band shifts are based on the orientation of the analyte relative to the substrate. Therefore, a portion of molecules may be affected by the field [74,75]. Zong et al. reported that certain peaks at 1079, 1137, 1287, and 1383 cm^−1^ are specifically found in CKD patients and can be associated with excess protein in composition [25]. The obtained Raman peaks at 1091, 1144, and 1292 cm^−1^ are in proximity to these values. We assume that the identified peaks may be indicative of proteinuria in general and albuminuria in particular. The peak at 1497 cm^−1^ has been assumed to be present in control human urine samples [76].

We mentioned in the Section 3 that for the PCA-LDA, PLS-LDA, and PLS-SVM methods, the datasets were divided into training and testing sets in a ratio of 70:30. We found very similar classification performance between the leave-one-out and k-fold cross-validation approaches (5-fold and 10-fold). For instance, all three of them had mean AUCs, obtained by averaging the AUCs of the three above-mentioned classification methods (PCA-LDA, PLS-LDA and PLS-SVM), in the range of 0.761–0.768. However, we found the best correlation (0.92) between EFs for each of the three gold-film-based substrates, which were measured with 4-nitrothiophenol (4-NBT), as described later in the paper, and obtained with 10-fold cross-validation values of AUCs for each substrate. Therefore, hereafter, we focus on the results obtained with 10-fold cross validation, but we also report classification figure-of-merit (FOMs) for the 5-fold cross-validation and leave-one-out methods in the Supplementary Materials.

The summary results of the classification performance valuation using the PLS-LDA method for the best-performing set, namely, 200 nm diameter (D200 nm) Au NPs on the Au substrate at an albumin threshold of 0.3 g/L, are shown in Table 2. The table includes sensitivity, specificity, accuracy, and confusion matrices. A confusion matrix is used to summarize the performance of a classification model. The matrix is categorized into four segments: true positives (TPs), true negatives (TNs), false positives (FPs), and false negatives (FNs). All these parameters for the six characterized substrates with up to 10 principal components (PC) components can be found in Tables S1–S5 in the Supplementary Information.

The clinical identification parameters are shown for all six tested substrates, with a 70:30 training/testing split, in Table 3, and the same split ratio has been described in multiple publications [82,83,84]. In general, this table demonstrates that PLS-LDA showed slightly better performance across almost all substrates, with the exception of nanorods on silver. For instance, the AUC average for all six substrates decreases in a row 0.79 to 0.76 and 0.74 for PLS, PCA, and SVM, respectively. However, there is a small difference in terms of average accuracy (0.74, 0.73, 0.73) and average F-score (0.75, 0.74, 0.74) in the same row for PLS, PCA, and SVM. The nanoparticles and nanorods on gold film demonstrate much better classification parameters relative to the same parameters obtained for other substrates (Ag, Al, Si). For instance, the most of average AUCs for the three methods (PLS, SVM and PCA) on gold film (0.956, 0.862, and 0.755) are much larger than most of the average AUCs calculated on the other three substrates (0.765, 0.693, and 0.537), while the difference between the three average AUCs for the gold-based substrates and the three average AUCs for the non-gold-based substrates is significant (86% vs. 67%).

The best-performing substrate was D200 nm spheroidal AuNPs on gold film, followed by λ650 nm AuNRs on gold film (Au_650rods_AuNPs), which is serving as the second best-performing substrate. These two substrates demonstrated higher average AUCs (0.96 and 0.86, respectively) than any other substrate in the range of PCs indicated in Table 2 from 1 to 10. D200 nm AuNPs on Au were found to achieve 100% sensitivity, 92% specificity, and 96% accuracy using the PLS-LDA method. The λ750 nm AuNRs on the Au also performed well with respect to both methods and ranked third in classification performance for the studied datasets (with AUC values of 0.767 for PLS-LDA, 0.792 for PCA-LDA, and 0.706 for PLS-SVM). The average AUC, calculated as an average of the AUCs of all three tested methods (PCA, SVM and PLS), demonstrates nearly but not exactly the same trend, with a maximum value of 0.956 for D200 nm AuNPs on Au, followed by 0.862 for λ650 nm AuNRs on Au, 0.765 for D200 nm AuNPs on Al, and 0.755 for λ750 nm AuNRs on Au.

Although the top-performing substrate was gold, aluminum emerged as the second best-performing substrate. The performance of Au nanoparticles on aluminum foil (AUC: 0.765) was clearly better than that of nanorods on Ag (AUC: 0.693) and much better than that of nanorods on Si (0.537). These results confirm the strong potential of Al foil as a low-cost substrate for the detection of protein biomarkers by direct SERS, while other applications of Al foil as an SERS substrate, including sandwich immunoassays with SERS readouts, have already been reported in the literature [63,64,65,85].

**Figure 2:**
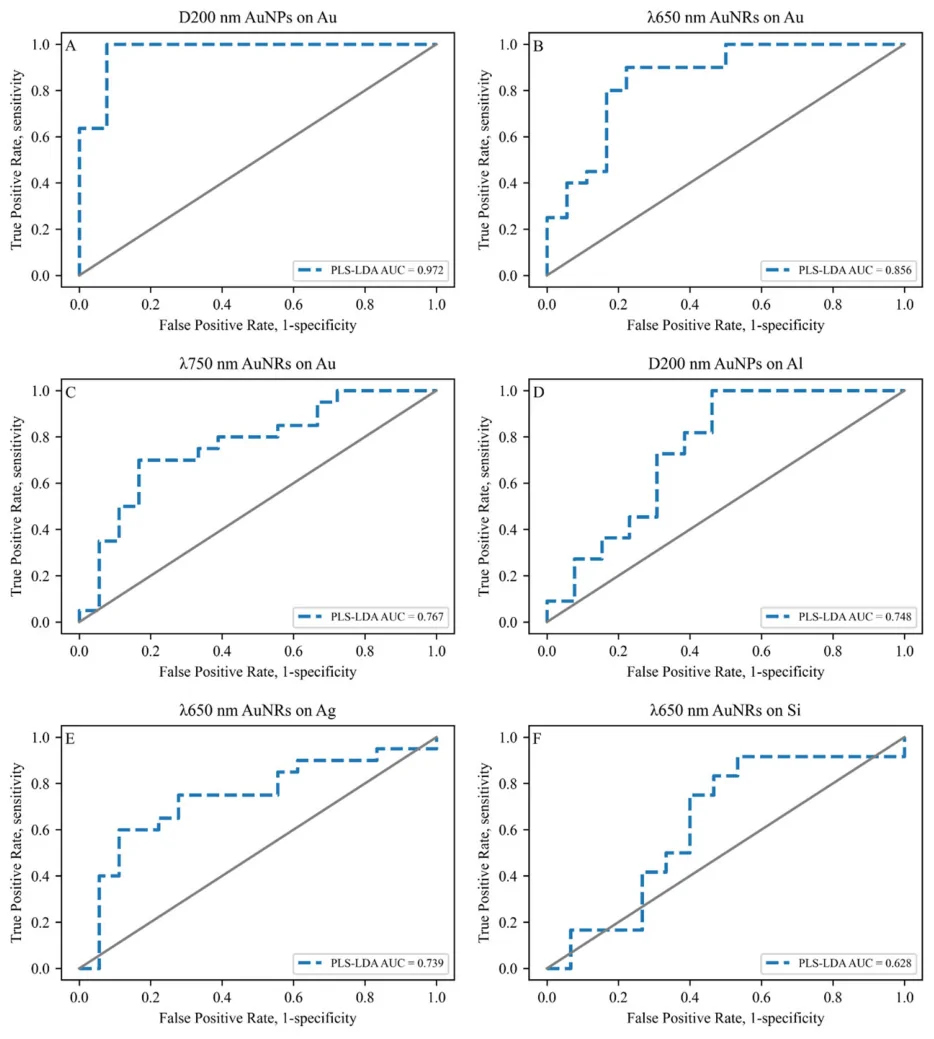
Receiver operating characteristic curves of human urine specimens: (**A**) 200 nm diameter (D200 nm) Au nanoparticles on Au substrate; (**B**) λ650 nm Au nanorods on Au substrate; (**C**) λ750 nm nanorods on Au substrate; (**D**) 200 nm diameter (D200 nm) nanoparticles on Al substrate; (**E**) λ650 nm Au nanorods on Ag substrate; and (**F**) λ650 nm Au nanorods on Si substrate.

The receiver operating characteristic (ROC) curves are shown for six sets (Figure 2) for the PLS-LDA model. The maximum area-under-the-curve (AUC) values were 0.972 for D200 nm Au nanoparticles on a Au substrate and 0.856 for λ650 nm Au nanorods on a Au substrate. The AUC value for the λ750 nm AuNPs on gold was 0.767, and the D200 nm gold nanoparticles on an Al substrate had an AUC value of 0.748. Figure 2 confirms that gold-film substrates have good classification performance and perform better than other non-gold substrates, and this figure visually demonstrates that the D200 nm spheroidal AuNPs on Au film exhibited better performance than that observed on any of the other (five) substrates.

**Figure 3:**
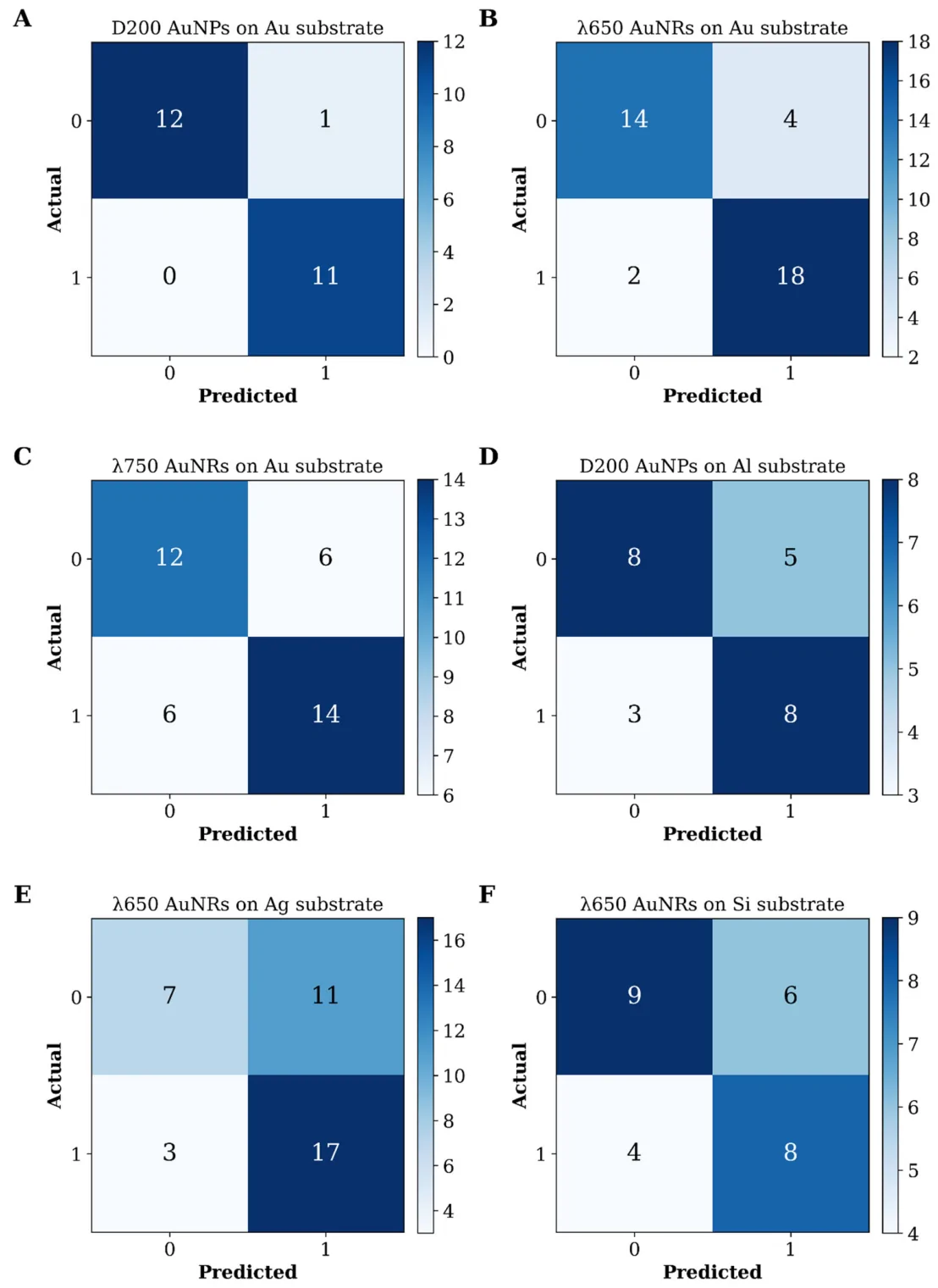
Confusion matrices of PLS-LDA classification results: (**A**) D200 AuNPs on Au substrate; (**B**) λ650 AuNRs on Au substrate; (**C**) λ750 AuNRs on Au substrate; (**D**) D200 AuNPs on Al substrate; (**E**) λ650 AuNRs on Ag substrate; and (**F**) λ650 AuNRs on Si substrate.

Figure 3 presents the confusion matrices of the PLS-LDA classification results for nanoparticles/nanorods on different substrates (Figure 3A–F), showing the distribution of true negatives (TNs) and true positives (TPs) along the diagonal, with comparatively fewer false positives (FPs) and false negatives (FNs), taken for the optimal number of PLS components. The confusion matrices of the PCA-LDA and PLS-SVM classification results are given in Figures S4 and S5 in the SI.

Overall, the performance of the λ650 nm AuNRs is better than that of the λ750 nm AuNRs. The reportted length and diameter of commercial λ650 nm AuNRs are in the range of 60–70 nm and 20–30 nm, respectively. The reported length and diameter of commercial λ750 nm AuNRs are in the range of 30–60 nm and 5–10 nm, respectively. A decrease in nanorod particle size may counterbalance the occurrence of stronger longitudinal surface plasmon resonance that we would expect for an excitation with a 785 nm laser [51]. Increasing nanoparticle size is expected to increase the electromagnetic decay length [60], which is likely to result in a more stable electromagnetic field near the surface with less dependence of SERS enhancement on the orientation of the protein molecule on the surface. Increasing nanorod size is likely to result in better access to hot spots. Gold nanorods display peak wavelengths of LSPR of nearly 650 nm and 750 nm when in suspension. In an ideal case, when we deal with a single nanorod or single nanoparticle, these λ max values should indicate better overlap with the 785 nm laser excitation wavelength, and we may expect greater SERS enhancement for both nanorods when compared to SERS enhancement on 200 nm diameter commercial gold nanoparticles, which have a peak wavelength of about 570 nm in suspension. Indeed, as shown in Figure 1, there is an about one-order-of-magnitude higher averaged Raman signal of urine on λ650 and λ750 nm nanorods in comparison to the same kind of averaged Raman signal on D200 nm AuNPs. However, the contrast between high- and low-albumin-concentration average spectra is better/greater for D200 nm AuNPs on gold film than for nanorods on gold or any other tested substrate (Ag, Si, and Al). This can be illustrated by a comparison of the ratio of Raman intensities, such as the intensity of high–low difference spectra at 1355 (or 1359) cm^−1^ (the gray lines in Figure 1A,E, with a peak that can be attributed to tryptophane) divided by the average intensity of high- and low-albumin spectra at 1000 cm^−1^, which is a relatively strong Raman characteristic peak for urea and therefore a good internal standard. These ratios are 30% for D200 nm AuNPs on gold film and 6% for λ650 nm nanorods on gold film, which means that the difference in albumin concentration is significantly more noticeable/better contrasted with D200 nm AuNPs than with λ650 nanorods.

**Figure 4:**
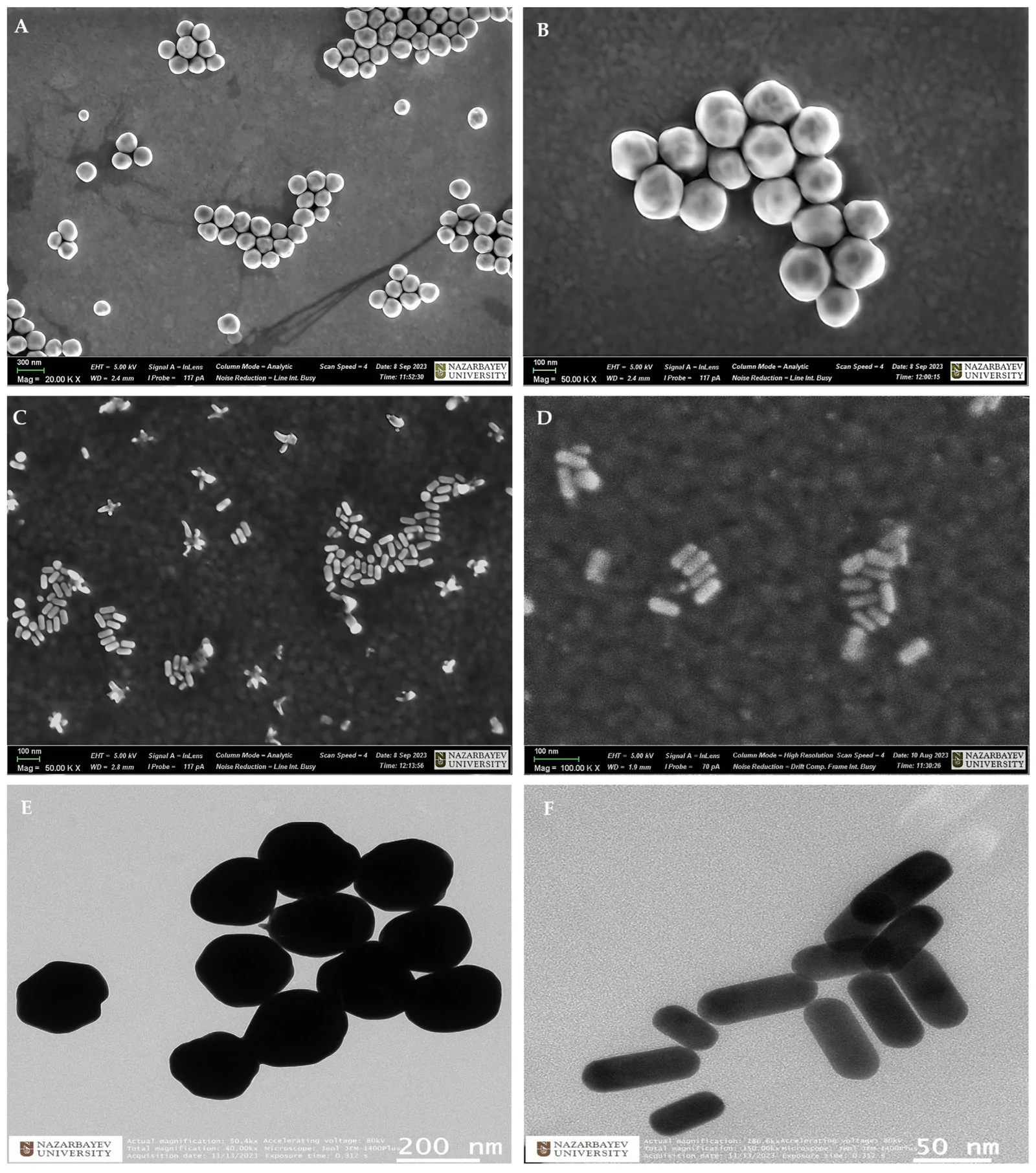
SEM and TEM images of 200 nm diameter (D200 nm) Au NPs on the gold substrates and λ650 nm Au NRs on silver substrates. (**A**) SEM image of D200 nm Au NPs at 20,000× magnification. (**B**) SEM image of D200 nm AuNPs at 50,000× magnification. (**C**) SEM image of λ650 nm Au NRs on silver substrates at 50,000× magnification. (**D**) SEM image of λ650 nm Au NRs on a silver substrate at 100,000× magnification. (**E**) TEM image of D200 nm Au NPs at 40 k× magnification. (**F**) TEM image of λ650 nm Au NRs at 150 k× magnification.

The Scanning Electron Microscope (SEM) and TEM images in Figure 4 demonstrate that the distribution and orientation of both the gold nanorods and commercial gold nanoparticles are far from uniform, and most of the nanorods exist on the surface of the substrate in oligomers. The scanning electron microscope images demonstrate that the average particle diameter and standard deviation of commercial D200 nm gold nanoparticles are 214 nm and 12 nm, respectively. We observed that the gold nanoparticles formed agglomerates. These nanoparticles do not have an ideal spherical shape, as they may resemble dodecahedrons. The TEM pictures show that the λ650 nm nanorods had an average length and width of 60.9 ± 4.3 nm and 25.5 ± 2.6 nm, respectively. The total quantities of particles were counted. By analyzing the TEM images shown in Figure 4E,F, we can find multiple cases of stacking of λ650 nm Au NRs where two layers and, a couple of times, even three layers of nanorods formed oligomers on the surface. A similar trend, where there is stacking for λ650 nm nanorods but no stacking for D200 nm AuNPs, is also demonstrated in Figures S7 and S8 in the Supplementary Information.

The formation of nanoparticle agglomerates is likely to boost the enhancement in SERS signals, while many SERS hot spots between nanoparticles will be created [86]. Stacking is likely to create a large number of hot spots, which may also be responsible for the relatively high SERS signal observed on the λ650 nm Au NRs in comparison to the signal observed on the D200 nm AuNPs. However, this three-dimensional distribution of hot spots for nanorods may reduce the reproducibility of the SERS signal due to the increasing variability in gap distance, gap area, and gap shape/orientation, which govern the enhancement in these hotspots. Another limitation of these two-layer aggregates of gold nanorods is impedance for the diffusion of protein molecules inside of these aggregates, which would create large variability in the SERS enhancement for larger molecules like proteins. Even single-layer nanorod agglomerates/oligomers have visibly much more narrow gaps between nanorods relative to the gaps visible in the TEM and SEM images of agglomerates/oligomers of the D200 nm AuNPs. Therefore, many relatively large protein molecules would be unlikely to diffuse inside and get into these hot spots created by nanorods, while smaller molecules like urea are more likely to diffuse into these hot spots and would experience much higher enhancement. Indeed, as shown in Figure 1A,C, the average characteristic peak of urea ~1000 cm^−1^ is much more intense on λ650 nm AuNRs in comparison to the same peak obtained on D200 nm AuNPs (with a ratio of roughly 1000 to 30). However, the hot spots created by D200 nm AuNPs should be, on average, much larger than hot spots created by nanorods, and D200 nm AuNPs are aggregated only in one layer. Therefore, they should be, on average, more accessible to protein or other larger molecules in comparison to most of the hot spots created by nanorods that are at least several times smaller, which, on average, are even less accessible due to stacking of some NRs in two or even three layers. Indeed, the relative signal intensity for amide bonds (range: 1241–1292 cm^−1^) for the average (SERS high–SERS low) spectrum, which is shown as a green line in Figure 1C, is generally higher for the spectrum measured on D200 nm AuNPs in comparison to the same kind of average intensity spectra shown as green lines in the spectra of nanorods (Figure 1A,B). The same factors would contribute to better clinical SERS performance of λ650 nm AuNRs in comparison to the performance of λ750 nm AuNRs when the 2–4-times-larger nanorods are likely to have larger hot spots, which, on average, are more accessible to protein molecules in comparison to those in the smaller nanorods.

Therefore, the diagnostic performance parameters (accuracy, AUC, etc.) of nanorods in the direct SERS assay of urine for albuminuria are good but not as good as the performance parameters in the assay with D200 nm gold nanoparticles. This observation is supported by the relatively high relative standard deviations in the measurements of the NBT signal for the calculation of the enhancement factors observed for the different substrates: D200 nm AuNPs on gold film show on average more reproducible distribution compared to λ650 and λ750 nm AuNRs on gold film (24% RSD vs. 21–38% RSD). Achieving a more uniform distribution of nanoparticles on a substrate with no stacking is bound to improve reproducibility and ultimately improve the results of the analysis.

Using SEM images, we calculated the number of λ650 nm and λ750 nm nanorods per square micrometer. This calculation showed a slightly higher (by <20%) surface concentration of larger λ650 nm nanorods (27 vs. 23 AuNRs per 1 µm^2^), which may slightly contribute to the better performance of the λ650 nm nanorods relative to the λ750 nm nanorods. However, there is a nearly 300% (×4 fold) higher EF for the same concentration of NBT when we compare the λ650 nm nanorods vs. the λ750 nm nanorods (both on gold film) as a substrate for NBT SERS measurement. Apparently, the larger diameter/size of λ650 nm nanorods is the most plausible explanation for the higher EF and for the better albuminuria-sensing performance as a result of it, with maximum AUCs, obtained with PLS, of 0.856 vs. 0.767. There are more TEM images of the λ650 nm AuNRs and D200 nm AuNPs in the Supplementary Materials.

The TEM and SEM images demonstrate apparently larger interparticle gaps for the D200 nm nanoparticles in comparison to the gaps between the λ650 nm AuNRs, which may indicate a greater possibility for protein molecules to diffuse into the hot spots, where high SERS EFs are achievable. Also, since 200 nm diameter nanoparticles are up to eight times taller relative to 25 nm diameter AUNRs, they are likely to create a larger gold surface area per unit of substrate area, where practically any protein molecules, including albumin molecules, can be absorbed and contribute to the SERS signal. Finally, the SEM and even TEM images of the λ650 nm AuNRs, as shown in Figure 4F, occasionally demonstrate nanorods stacked in two layers, which is likely to generate an even lower proportion of hot spots between nanorods available for proteins to diffuse into them, while there is practically no second layer of 200 nm AuNPs in the TEM or even SEM images.

**Figure 5:**
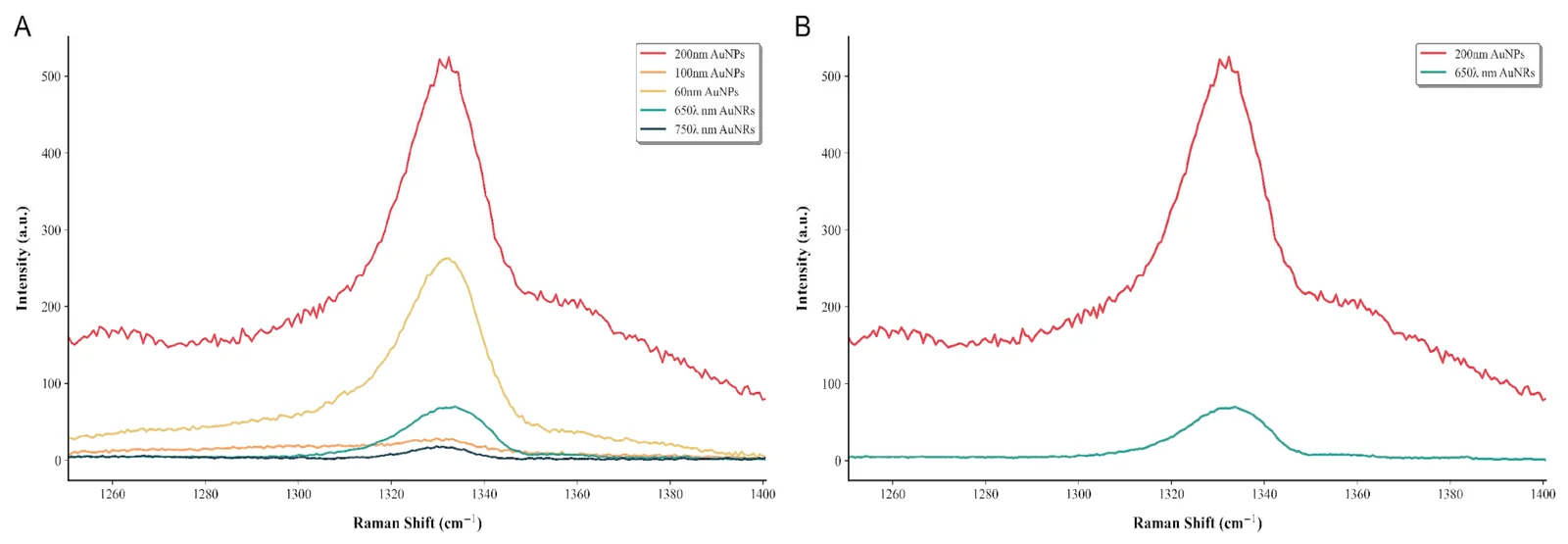
The characteristic signals of 4-NBT for (**A**) 60 nm, 100 nm, and 200 nm AuNPs; 650 nm AuNRs; and 750 nm AuNRs on gold substrates. (**B**) This figure shows 200 nm AuNPs and 650 nm AuNRs on gold substrates.

The enhancement factor (EF) of the gold-film-based substrates in the presence of 4-nitrothiophenol (4-NBT) was studied, as described in Section 3 later. The process of EF calculation and the results are also described in detail in the Supplementary Materials, including Tables S8–S10 and Figure S6, which are based on relevant references [85,87]. The highest EF was found to be 5.42 × 10^7^ for D200 nm AuNPs on a gold substrate. The EF results for the λ650 nm and λ750 nm AuNRs are 1.12 × 10^7^ and 2.71 × 10^6^, respectively. The SERS spectra of 4-NBT on different NPs on gold substrates are illustrated in Figure 5.

**Figure 6:**
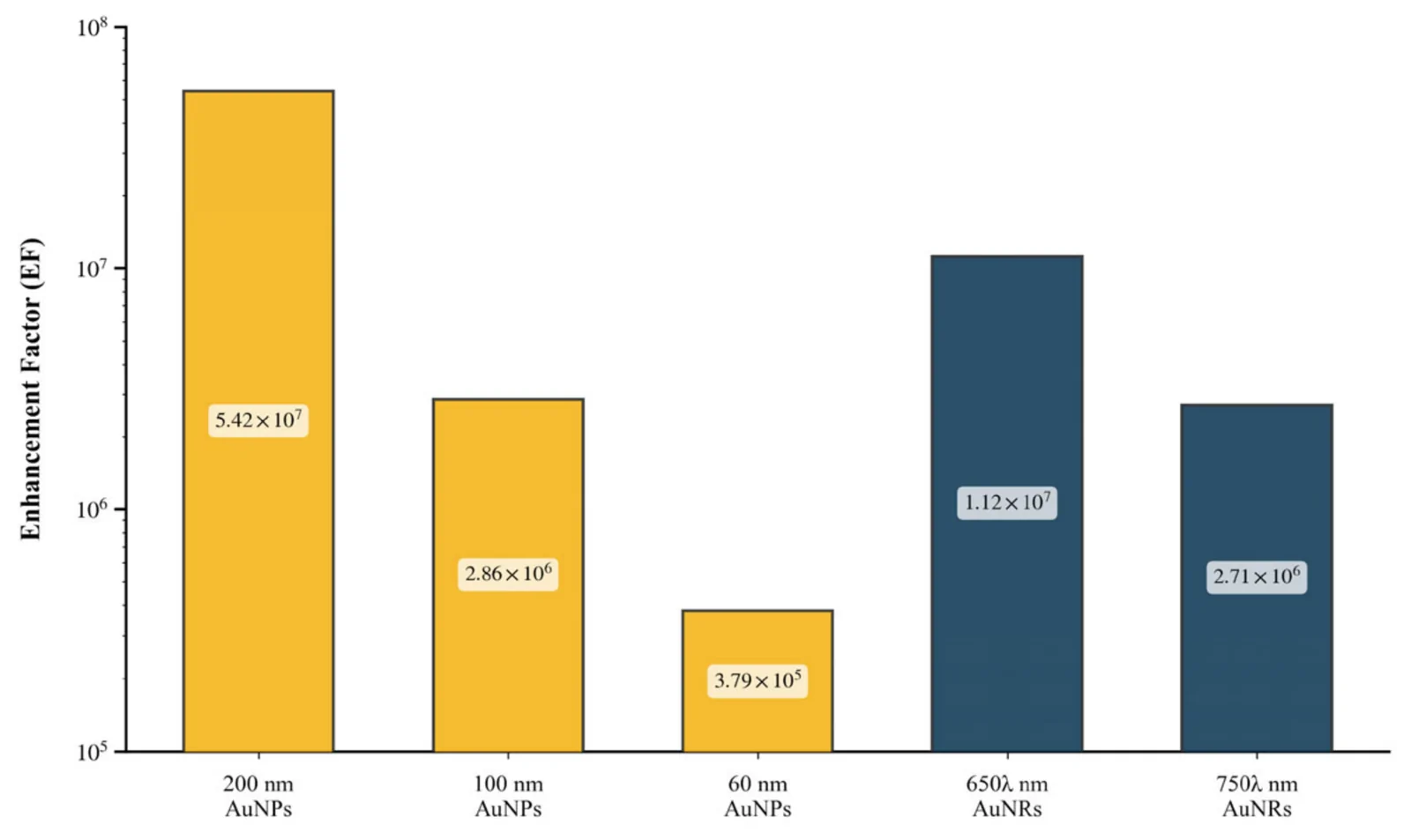
A bar chart of SERS EFs, calculated for NBT with different nanoparticles/nanorods on gold-film substrates.

The bar chart below shows EF results for different NPs on gold substrates (Figure 6). As expected, the EF for 60 nm AuNPs was lower (3.79 × 10^5^) compared to that for the 100 nm AuNPs (2.86 × 10^6^). The observed EF for 200 nm diameter AuNPs exceeded the calculated EF for 100 nm AuNPs by a factor of almost 20 (54 × 10^6^ vs. 2.9 × 10^6^), while the shapes of both nanoparticles are similar, and the only essential difference (two-fold) is in diameter. The largest nanoparticles (D200 nm) that we tested as a substrate demonstrated both by far the highest SERS EF among all tested nanoparticles and the highest maximum AUC in high-albumin-content screening of urine (0.972).

In general, we observed a strong correlation (0.92) between nanoparticle/nanorod albuminuria-sensing performance, taken as the maximum AUC and EF calculated for a 10 nM solution of NBT from measurements done on the same substrates (nanoparticles or NRs on gold film), when in a sequence: for D200 nm AuNPs, λ650 nm AuNRs, and λ750 nm AuNRs, the average maximum AUC decreases were 0.956, 0.862, and 0.755, and EFs also decreased by 54.2 M, 11.2 M, and 2.9 M (M is 10^6^), respectively. The calculated correlation coefficients of AUC vs. EF for all three cross-validation methods and the relative standard deviations of the AUC for the three top-performing substrates (all on gold film) are demonstrated in Table S7 of the Supplementary Materials.

Compared with other reported SERS studies, our best-performing substrate, 200D AuNPs on Au, demonstrates comparable or better classification performance (Table 4). The 200D AuNPs on Au achieved the highest AUC (0.97) and accuracy (0.96), exceedingly most reported AgNP-based substrates. Notably, the 200D AuNP substrate exhibited a high enhancement factor (5.4 × 10^7^), whereas enhancement factors were not reported in the other studies listed.

Overall, in the present publication, we obtained measurements from a total of 614 samples on six substrates, or a total of 11,052 spectra of urine samples, and we also calculated EFs for NBT on five substrates, including three best-performing substrates applied in a current publication, which are shown in Table 4 above.

## 3. Materials and Methods

### 3.1. Urine Sample Collection

The urine specimens used in this study were second-morning urine samples. Each specimen container was labeled with the patient’s identification details before collection. Patients were recruited by the hospital, and the corresponding clinical laboratory data were provided for all samples. For the gold nanorod dataset, 124 urine samples were included, comprising 60 samples with albumin concentrations < 0.3 g/L and 64 samples with albumin concentrations > 0.3 g/L. For the D200 nm gold nanoparticle dataset, 77 urine samples were included, comprising 42 samples with albumin concentrations < 0.3 g/L and 35 samples with albumin concentrations > 0.3 g/L.

Urine albumin concentrations were determined in the hospital laboratory via a quantitative immunoturbidimetric assay using the Cobas 6000 system (Roche Diagnostics, Mannheim, Germany). In this study, these urine albumin results were used as the clinical reference for urinary protein status. Additional urinary chemical parameters provided by the hospital were also recorded. After collection, urine samples were stored at −20 °C until analysis.

### 3.2. Reagents and Lab Equipment

Gold nanorods with a surface plasmon resonance (SPR) peak of 750 nm and a reported diameter range of 5–10 nm were purchased from Nanocs Inc. (Boston, MA, USA). Au NRs were used as-received, with a reported gold concentration of 0.01% (weight per volume). 4-Nitrothiophenol (4-NBT) and gold nanorods with SPR 650 nm with a diameter range of 20–30 nm and 200 nm gold nanoparticles were purchased from Sigma-Aldrich (St. Louis, MO, USA). Reported concentrations of Au NRs and 200 nm Au NPs were 45 μg/mL in water and 1.9 × 10^9^ particles/mL, respectively. Gold and silver slides (substrates) were supplied by Evaporated Metal Films (EMF) Dynasil (Omega Optical, Brattleboro, VT, USA). Raman images were acquired with Raman Spectroscopy LabRAM HR Evolution (Horiba-France, Villeneuve d’Ascq, France).

### 3.3. SEM Parameters

SEM images were acquired using a Zeiss Crossbeam 540 scanning electron microscope (Oberkochen, Germany). SEM analysis parameters were as follows: electron high tension (EHT) = 5.00 kV, working distance (WD) = 5.1 mm, probe current = 139 pA, and Energy-Selective Backscattered (ESB) Detector Grid Voltage = 833 V.

### 3.4. TEM Parameters

TEM images were captured using a JEOL JEM-1400 Plus transmission electron microscope (Tokyo, Japan). TEM analysis parameters were as follows: magnification = 40,000–150,000×, exposure time = 1.25 s, and accelerating voltage = 80 kV.

### 3.5. Preparation of Urine Samples on Substrate

**Figure 7:**
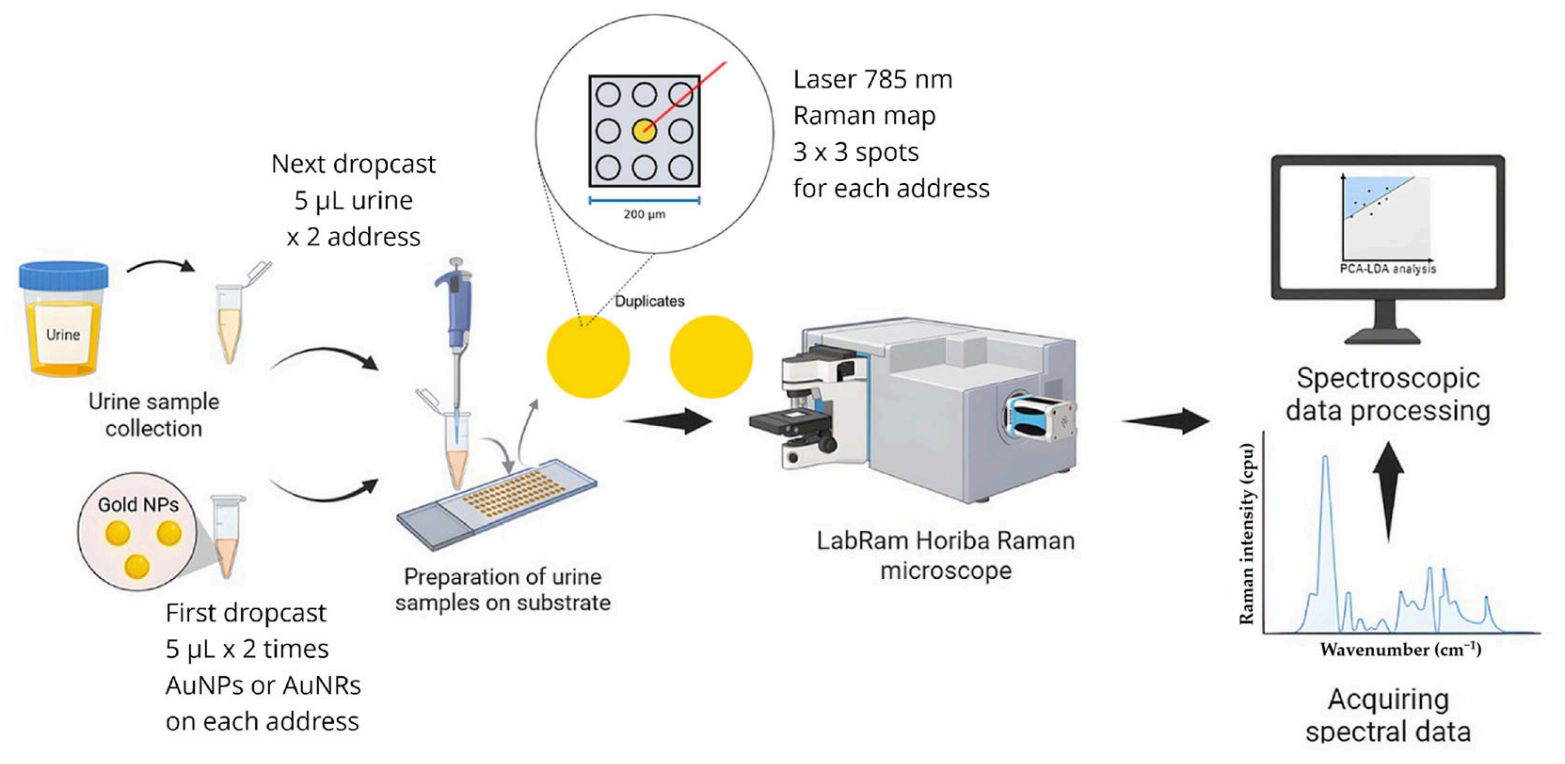
Workflow of urine sample analysis for Raman measurements.

The workflow of the urine sample analysis starting from substrate preparation is schematically illustrated in Figure 7. The preparation process, in principle, is like the preparation of commercial gold nanoparticles on gold and silver substrates reported previously [33,85,90]. First, λ650 nm and λ750 nm gold nanorods and 200 nm diameter (D200 nm) nanoparticles were poured into four tubes, with a volume of 1.2 mL each. Then, λ650 nm and λ750 nm nanorods were centrifuged for 5 min at 8000 rpm and 10,000 rpm, respectively, and the supernatant was extracted (about 1.1–1.15 mL) and resuspended in deionized (DI) water. D200 nm NPs were centrifuged and resuspended in DI water twice for 5 min at 2500 rpm. We applied a lower volume of the prepared-by-×2 centrifugation/resuspension-cycle nanoparticle suspension (5 μL) instead of the 15 μL, which was a previously used volume of the suspension [90]. Then, 5 microliter (µL) aliquots from the prepared 1.8 milliliter total (mL) solution of gold nanorods were droplet-casted on the gold and silver substrates under normal room conditions and air-dried. Then, another 5 µL of gold nanorods was dropped on the slides and left to dry naturally. D200 gold nanoparticles were droplet-casted onto the gold surface. We applied a lower volume of nanoparticles—5 μL. Urine samples were thawed after storage in the freezer. Then, 5 µL of urine samples was drop-casted onto the spots on solid substrates obtained from all nanoparticles. The urine specimens were thawed after being stored in a freezer at −20 °C. Each sample was prepared and measured in duplicate.

### 3.6. Preparation for EF Calculations

A 10 nanomolar (nM) 4-nitrothiophenol (4-NBT) solution (molecular weight: 155.17 g/mol) was prepared, and 5 μL aliquots were drop-casted onto the gold substrate, resulting in spots measuring approximately 3 mm in diameter. A detailed description of the EF calculation method is provided in the Supplementary Information.

### 3.7. Acquiring Spectral Data

Raman measurements were conducted using a LabRam Horiba microscope (Horiba-France, Villeneuve d’Ascq, France) with the following parameters: laser excitation wavelength, 785 nm; spectral range, 970–1800 cm^−1^; diffraction grating, 600 g/mm; laser power, 29 mW; acquisition time, 30 s; objective, ×10; and map size, 3 × 3. For each droplet, 9 spectra were measured; hence, 18 spectra were measured for each specimen. For enhancement factor calculations, the same Raman parameters as described above were applied, except for the following: a spectral range of 1100–1600 cm^−1^, an acquisition time of 4 s, a map size of 2 × 2, and 4 spots per map, measuring a total of 16 spectra. The SERS signal of 4-NBT was quantified via the N-O stretching vibration detected at 1336 cm^−1^, in agreement with the reported literature value at 1335–1336 cm^−1^ [85,91,92].

### 3.8. Processing of Spectroscopic Data

Spectroscopic data were processed in Python 3.13.9, with Jupyter Notebook 7.4.5 used as a programming interface. Multiple studies have implemented median filtering and the Savitzky–Golay technique for spectral preprocessing [93]. In this work, we also applied these methods after asymmetric least squares (ALS) baseline correction to separate the true signal from background noise, excluding fluorescent background, a method that is commonly reported [94,95]. Median filtering removes outliers, and the Savitsky–Golay smoothing algorithm smooths the desired signal [93,96]. Therefore, the sequence that we use in spectral-data processing is ALS baseline correction first, then median filtering to remove outliers, and finally the Savitzky–Golay method. Examples of spectra before and after background correction are given in the Supplementary Information (SI). Prior to model development, the dataset was divided into a training set (70%) and an independent test set (30%) using stratified random sampling to preserve binary class proportions [82,83,84]. Three distinct classification pipelines, PCA-LDA, PLS-LDA and PLS-SVM, were employed to process spectroscopic data in order to analyze the differences between two groups. Both PLS and PCA were utilized as initial dimensionality reduction techniques to project the high-dimensional data into a lower-dimensional space of orthogonal components. The optimal number of latent variables for PLS-LDA and PLS-SVM models and optimal number of principal components for PCA-LDA were determined via different internal cross-validation methods on the training set, and the number of components that minimized the Mean Squared Error (MSE) was selected as optimal [97,98]. We performed leave-one-out cross-validation (LOOCV) on training set, in which each sample was used once as the validation set, while the remaining samples were used for model training [99]. In addition, to further evaluate the robustness of the models, k-fold cross-validation was investigated by following the widely adopted methodology reported by Buhas and Zhang et al. [100,101]. In k-fold CV, the data are partitioned into k equal parts (folds), and the model is trained on k-1 folds and tested on the remaining fold. This process is repeated k times; in practice, k is often taken to be 5 or 10. Among the evaluated cross-validation methods, all approaches provided close and, on average, very similar estimates of model performance. However, 10-fold cross-validation yielded a slightly better correlation of classification performance results for each particular substrate with SERS enhancement factors obtained/calculated for the same substrates. Therefore, we selected the results obtained with 10-fold cross-validation and report them in Table 2 and Table 3 and Figure 2 and Figure 3, discussing them throughout the manuscript. Other results obtained with LOOCV, and 5-fold cross-validation, are shown in the Supplementary Materials. Following dimensionality reduction, LDA was applied to the retained scores of the PCA-LDA and PLS-LDA models, while the PLS-SVM pipeline employed a Support Vector Machine (SVM) classifier using the retained PLS latent variables.

To optimize the decision boundaries, classification thresholds were established by maximizing Youden’s J statistic (the optimal balance between true-positive rate and false -positive rate) derived from the receiver operating characteristic (ROC) curves [102,103]. All mathematical modeling and statistical analyses were conducted in Python 3.13.9 utilizing the scikit-learn 1.7.2, pandas 2.3.3, and numpy 2.3.5 libraries [104]. To assess the reliability and performance of a statistical model, we calculated figures such as sensitivity, specificity, and accuracy. Sensitivity examines the ability of tests to determine positive diseases. Specificity indicates the way healthy controls can be accurately excluded. The ROC curve is an illustration of specificity and sensitivity. Accuracy estimates the correctness of diagnostic tests and can be calculated using sensitivity and specificity [105]. The F-score is a predictive performance metric for binary classification models that integrates precision and recall through their harmonic mean, providing a balanced assessment of classifier accuracy [106]. The following equations were used to calculate the parameters described above [101,105]:

$$\mathrm{Sensitivity}=\frac{\mathrm{Number of true positives}}{\mathrm{Number of true positives}+\mathrm{number of false negatives}}$$

$$\mathrm{Specificity}=\frac{\mathrm{Number of true negatives}}{\mathrm{Number of true negatives}+\mathrm{number of false positives}}$$

$$\mathrm{Accuracy}=\frac{\mathrm{Number of true predictions}}{\mathrm{Number of all predictions}}$$

$$F-\mathrm{score}=\frac{2*\mathrm{Number of true positives}}{2*\mathrm{Number of true positives}+\mathrm{number of false positives}+\mathrm{number of false negatives}}$$

## 4. Conclusions

Good clinical analysis figure-of-merit (FoM) values were achieved by implementing gold nanoparticles with a diameter of 200 nm (D200 nm) on gold film as well as λ650 nm and λ750 nm gold nanorods on gold and silver substrates, as evident from the maximum AUC ROC values. Nanorods on silver films demonstrated lower performance relative to the performance of the same nanorods on gold films (with a max. AUC of 0.739 vs. a max. AUC of 0.856), which may have occurred due to silver oxidation/corrosion in urine [107,108]. Also, this may have occurred due to the expected decrease in plasmonic coupling between the relatively redshifted plasmon of nanorods and silver films, which, in general, should have a more blue-shifted plasmon than gold film with respect to a laser excitation wavelength of 785 nm [109,110].

We found that λ650 nm gold nanorods yielded better results than λ750 nm AuNRs on gold film substrates (with an average AUC of 0.862 vs. 0.693 for the three methods), which may be explained by the larger size of λ650 nm Au NRs (D 20–30 nm vs. D 5–10 nm) and therefore the larger size of hot spots between nanorods, which would be more available for protein adsorption, as well as the greater EMF decay length, which may provide more stable enhancement that is less dependent on the orientation of a protein molecule, such as an albumin molecule, near the nanorod or nanoparticle surface.

Among the various substrate configurations that we tested, the largest-diameter 200 nm (D200 nm) nanoparticles on gold demonstrated superior performance. It was previously reported that 100 nm gold nanoparticles, on average, produced slightly better results than 60 nm gold nanoparticles [62]. This trend continued, and we observed even more significant improvement in the clinical FoMs of SERS from D100 nm to D200 nm gold nanoparticles on gold film.

The best-calculated average sensitivity, specificity, accuracy, and AUC were 1.0, 0.92, 0.96, and 0.972, respectively, and they were obtained with D200 nm gold nanoparticles on gold film. This substrate is likely the most promising substrate for the detection of albuminuria, which is a specific but very common form of proteinuria, evaluated with direct SERS of urine.

One of the possible explanations for the better clinical performance of larger nanoparticles, relative to gold nanorods or smaller-diameter gold nanoparticles, may be an abundance of relatively large SERS hot spots. Such relatively large hot spots are likely to present greater availability for the diffusion of protein molecules, including albumin molecules, towards these hot spots in comparison to the diffusion of proteins towards hot spots formed, for instance, by relatively small nanorods.

Overall, SERS spectroscopy of urine showed significantly improved potential in distinguishing individuals with normal urinal concentrations from patients with elevated urinal albumin concentrations when new SERS substrates, which have never been previously tested for albuminuria or proteinuria identification, were applied. These well-performing substrates include nanorods and, in particular, relatively large 200 nm diameter gold nanoparticles on gold film. To the best of our knowledge, this is the first application of such a substrate, fabricated with relatively large-diameter (200 nm) AuNPs on gold film, for the clinical application of SERS. In addition, this work is a rare comparative study that analyzes two different morphologies (rods and spheroids) on a substrate and various materials (Au, Ag, Al and Si) for the substrate.

We plan to continue working on substrates for direct SERS detection of other biomarkers in urine, applying large gold nanoparticles and nanorods on gold film as well as other multilayer SERS substrates.

## Figures and Tables

**Table 1:** Noticeable peaks in Raman spectra of urine: band assignment according to references.

Urine Component	Structure	SERS Peaks in Urine from References, (cm^−1^)	Observed SERS Peaks (cm^−1^) in Urine Spectra
Albumin [72]	Complex molecule (protein)	1235, 1319 (Raman)	1249, 1304
Urea [73,74,75,76]	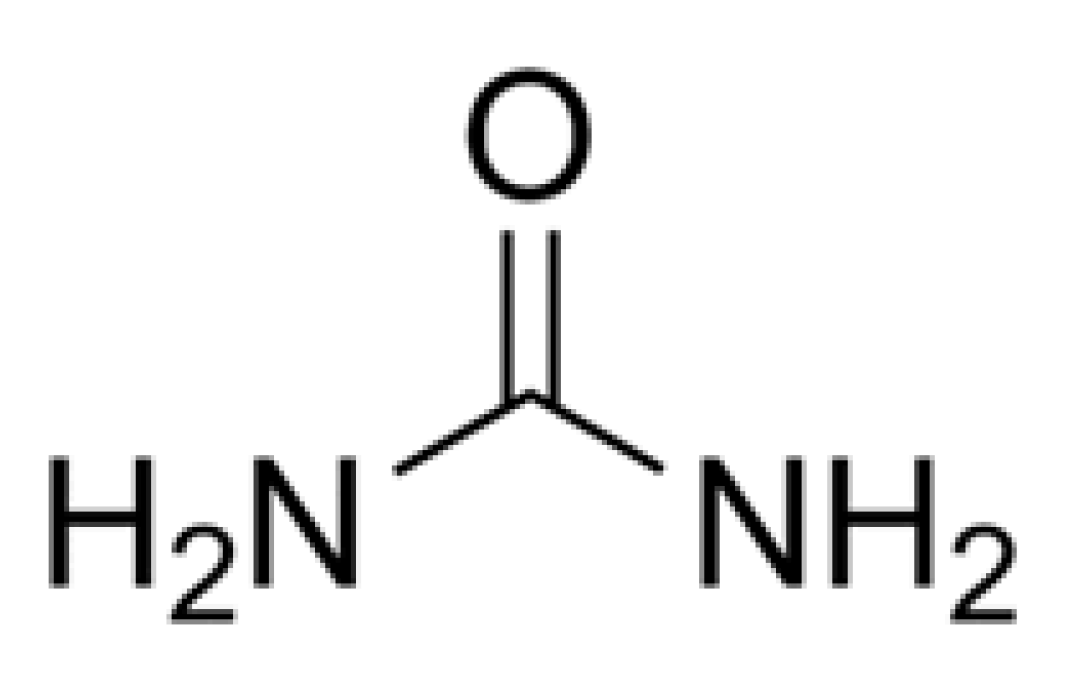	1000–1013, 1159 (Raman), 1576 (Raman)	1000–1004, 1157, 1560–1600
Tryptophan [74]	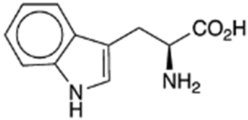	1358	1359–1363
Uric acid [77]	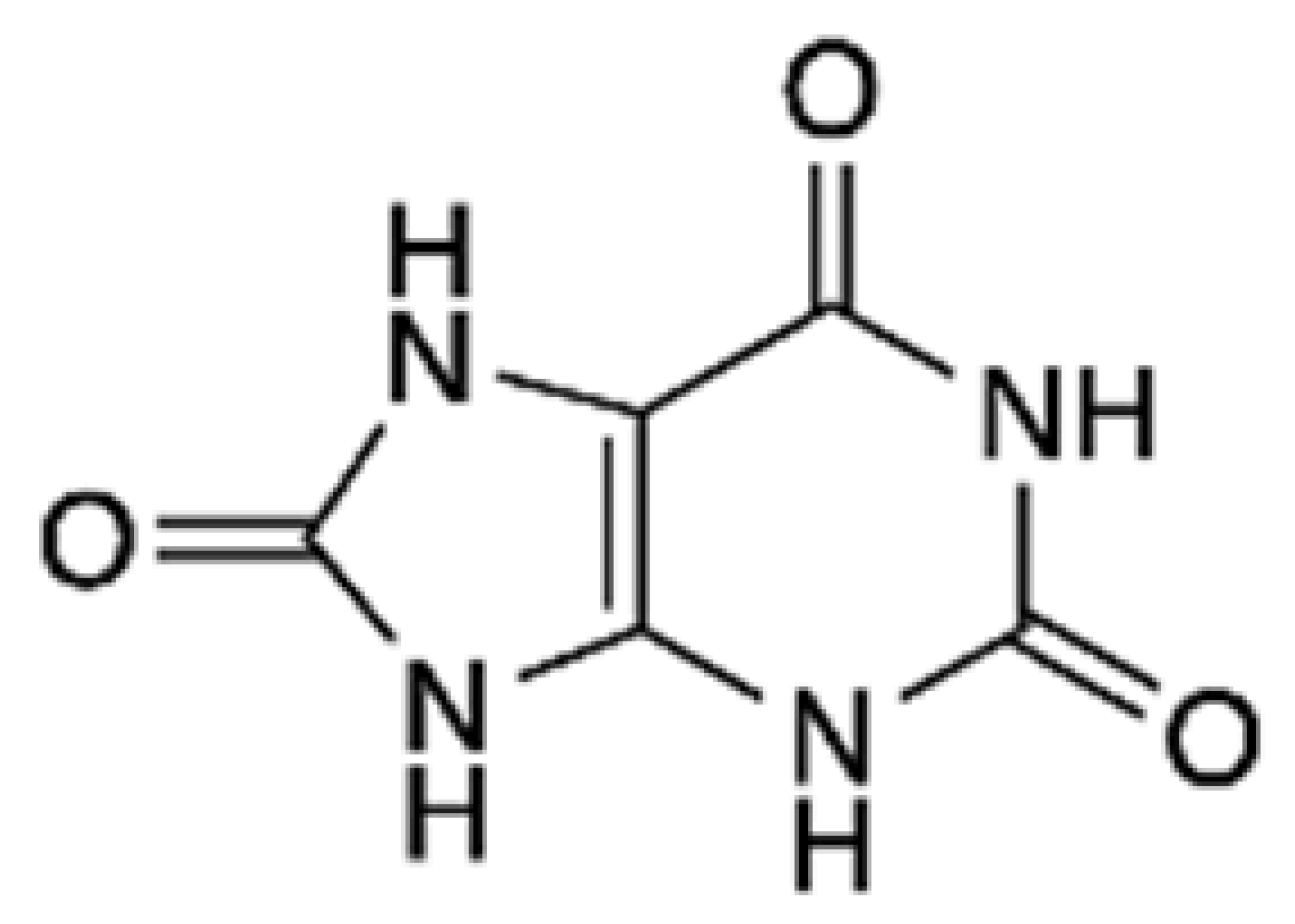	1436, 1595 (Raman)	1444,1598
Creatinine [72,74]	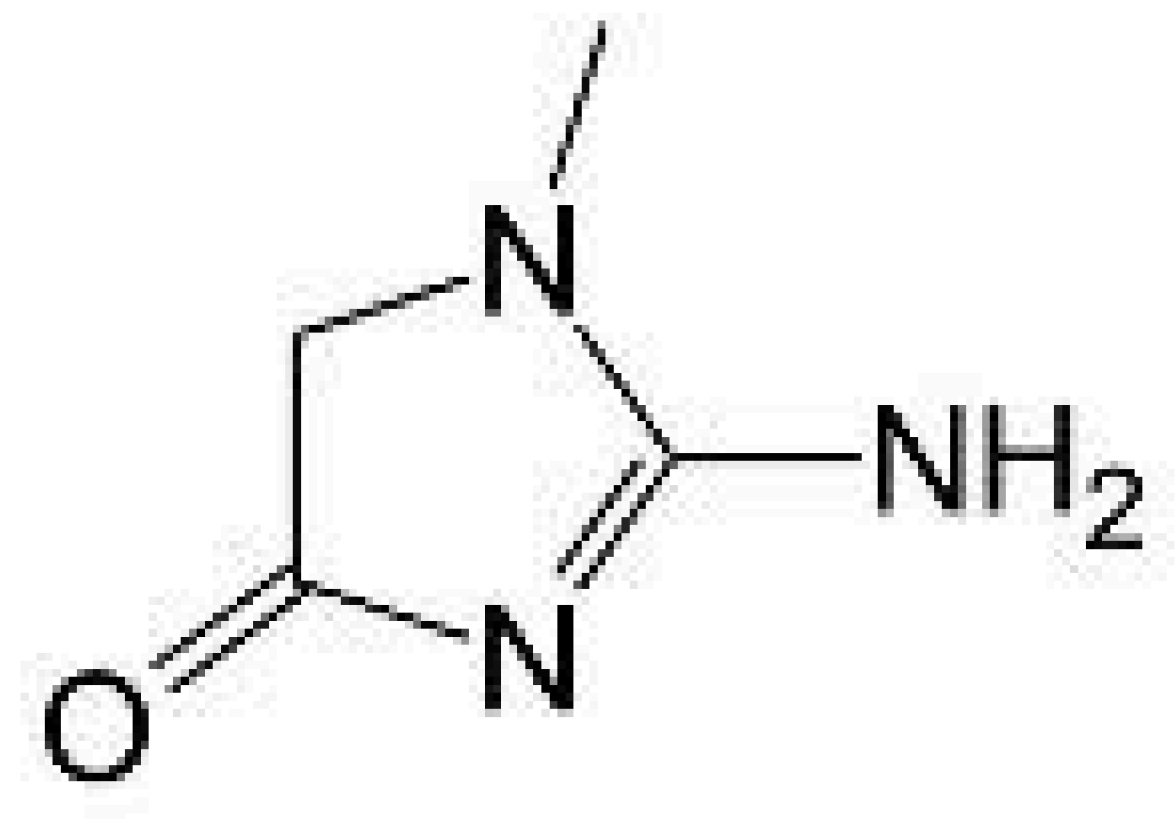	1397 (Raman), 1420–1426	1409, 1425
Creatine [76]	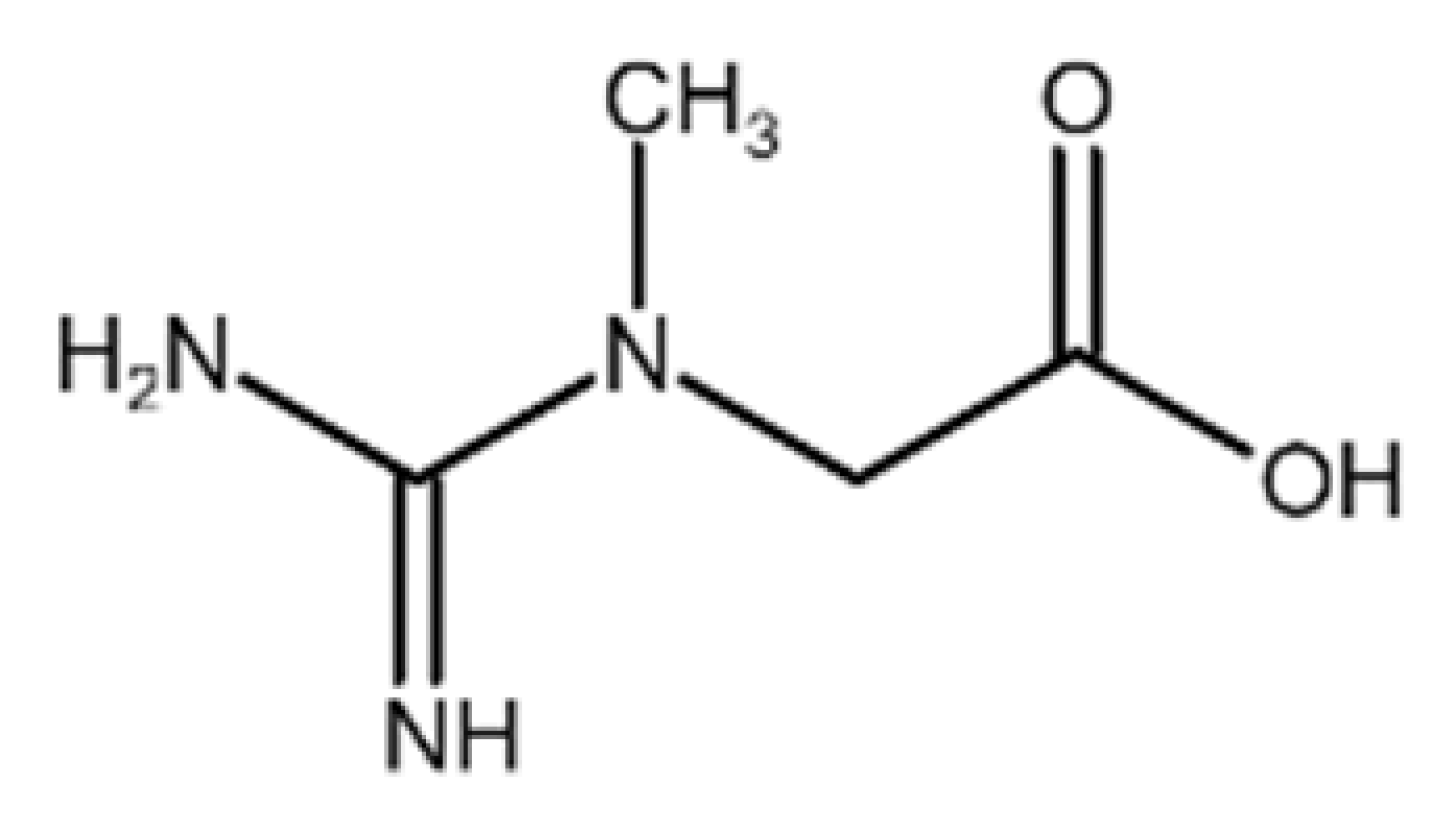	1397 (Raman)	1409
Water (H-O-H bending mode) [72]	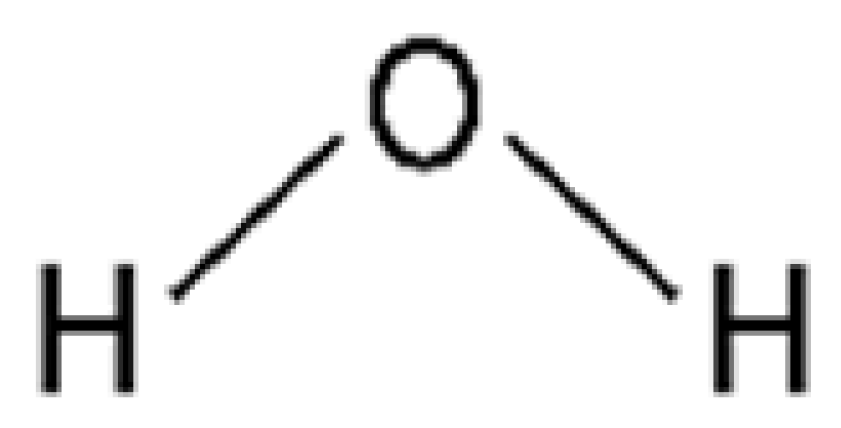	1349 (Raman)	1348–1355
Ketone bodies, such as hydroxybutyrate and acetoacetate [75]	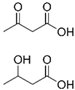	1456 (Raman)	1448–1449
Amino acids [78]	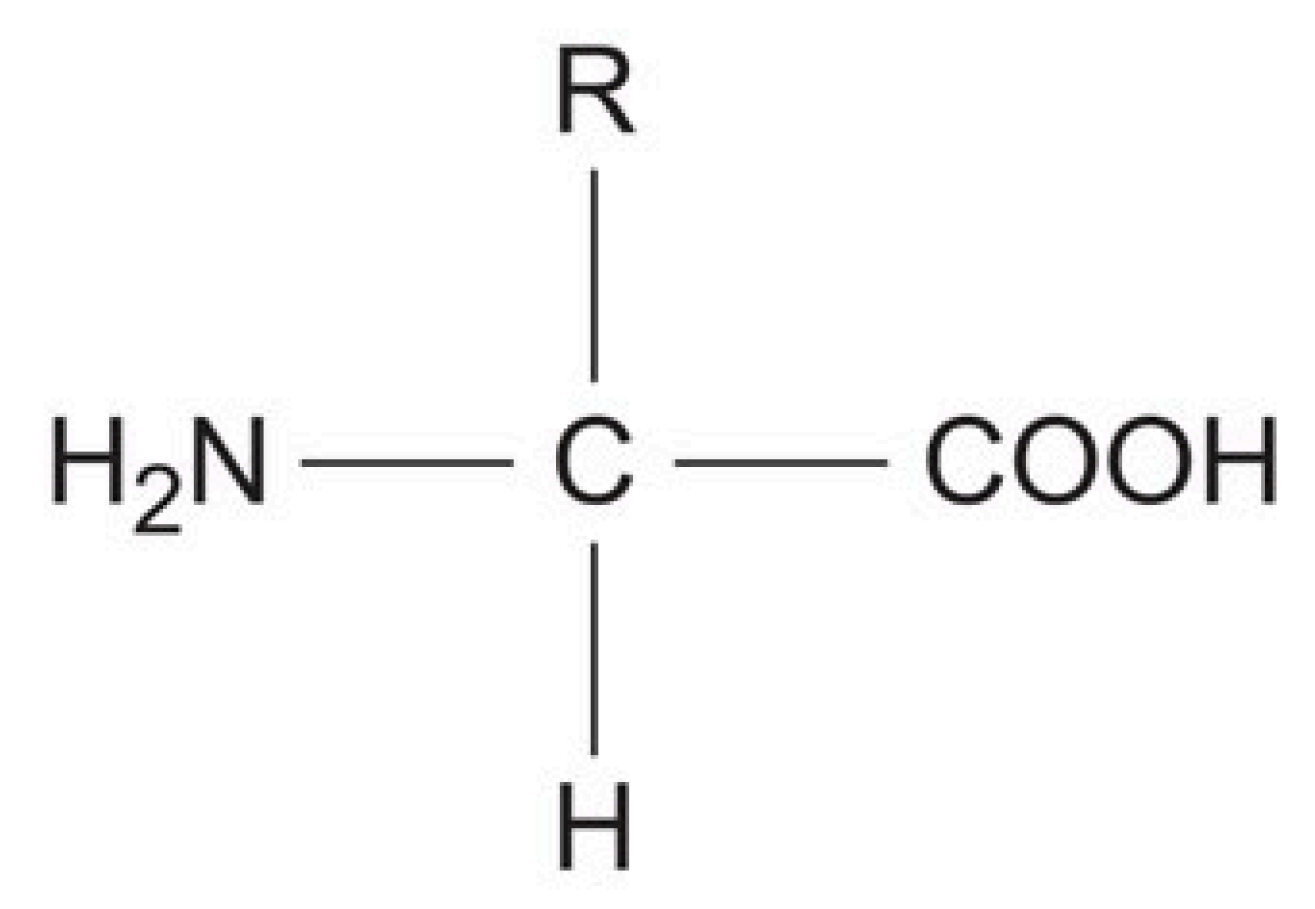	1703 (Raman)	1722
Carbohydrates (glucose) [79]	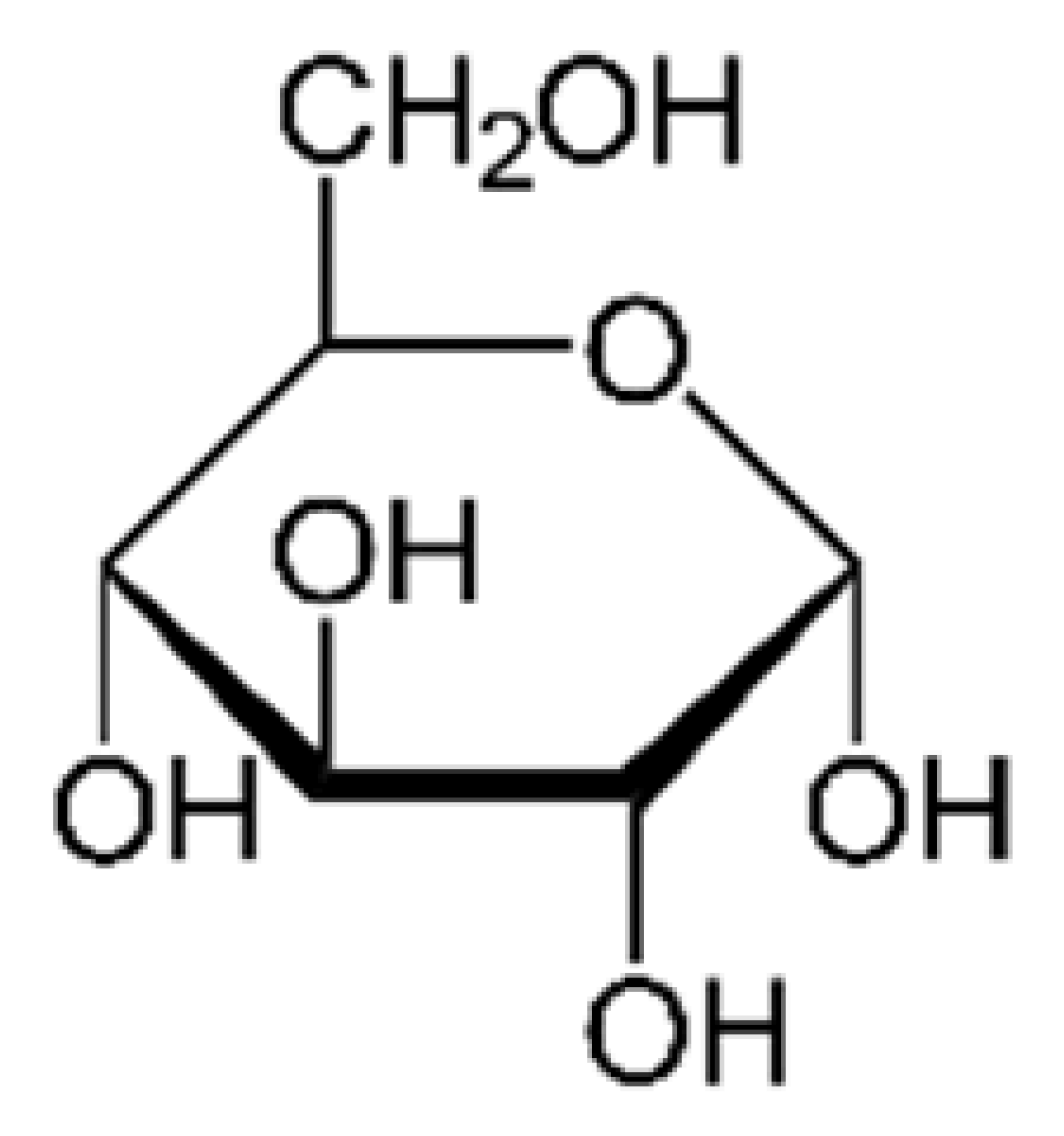	1177	1171
Amide III [80,81]	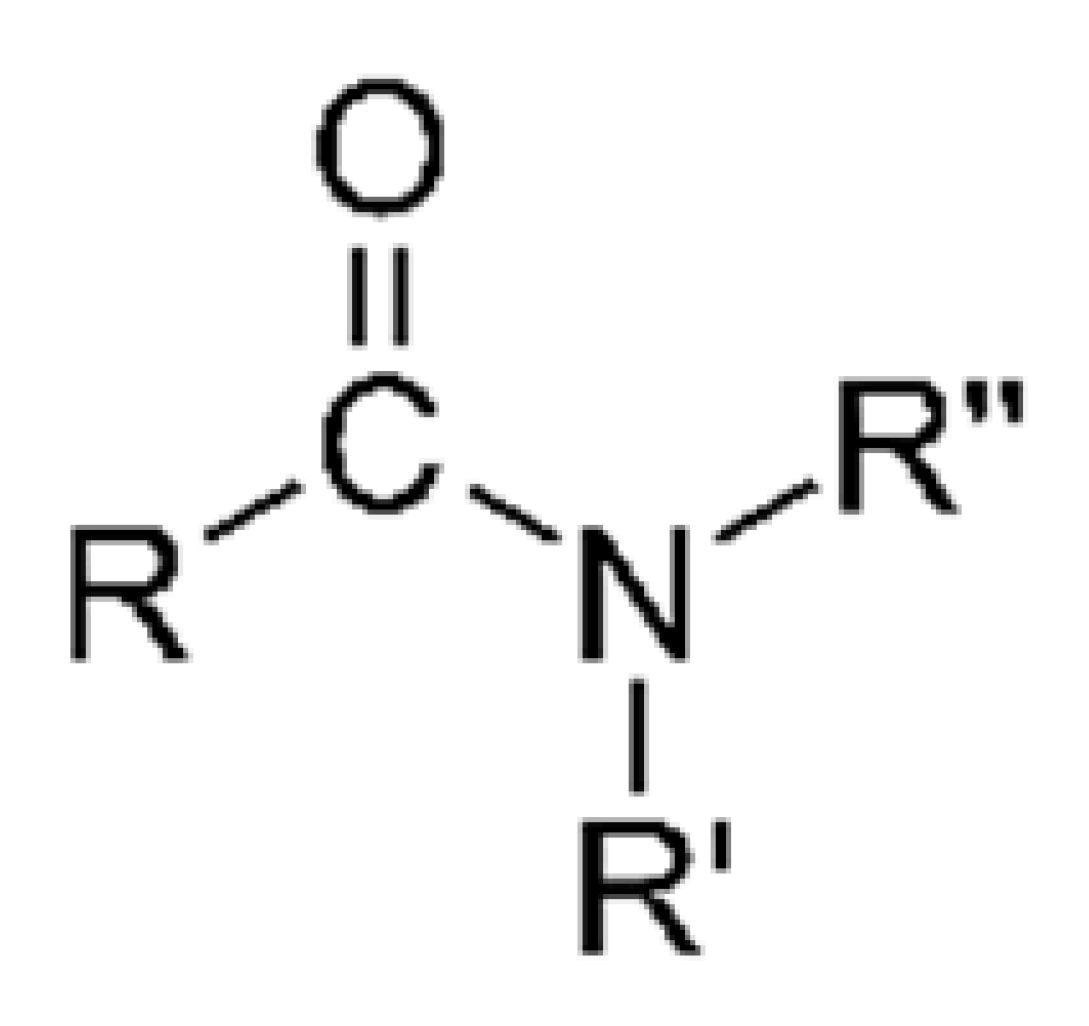	1239 (anti-mouse Immunoglobulin G ( IgG) and IgG-anti-IgG complex), 1200–1300 (Raman)	1241–1292

**Table 2:** Summarized tables of classification performance for the 200 nm diameter (D200 nm) Au NPs on a Au substrate for the PLS-LDA model. Albumin threshold was 0.3 g/L.

A	PLS Results for D200 AuNPs on Au				*N* Samples = 24			
Component	Sensitivity	Specificity	Accuracy	AUC	TP	TN	FP	FN
1	0.91	0.85	0.88	0.94	10	11	2	1
2	1.00	0.92	0.96	0.97	11	12	1	0
3	0.36	1.00	0.71	0.82	4	13	0	7
4	0.64	0.92	0.79	0.82	7	12	1	4
5	0.55	0.92	0.75	0.79	6	12	1	5
6	0.73	0.69	0.71	0.75	8	9	4	3
7	0.73	0.77	0.75	0.73	8	10	3	3
8	0.27	1.00	0.67	0.69	3	13	0	8
9	0.64	0.54	0.58	0.70	7	7	6	4
10	0.73	0.54	0.63	0.73	8	7	6	3

**Table 3:** A summarized table of classification performance for all substrates, with k-10-fold internal validation.

	70/30 Split						Average for 6 Substrates
	D200 nm AuNPs on Au	λ650 nm AuNRs on Au	λ750 nm AuNRs on Au	λ650 nm AuNRs on Ag	D200 nm AuNPs on Al	λ650 nm AuNRs on Si	
**Number of samples**	77	124	124	124	77	88	102
**Sensitivity for PLS**	1.00	0.90	0.70	0.85	0.73	0.67	0.81
**Sensitivity for PCA**	1.00	0.85	0.60	0.45	1.00	0.83	0.79
**Sensitivity for SVM**	0.91	0.90	0.80	0.60	0.73	0.67	0.77
**Specificity for PLS**	0.92	0.78	0.67	0.39	0.62	0.60	0.66
**Specificity for PCA**	0.85	0.78	0.89	0.89	0.54	0.20	0.69
**Specificity for SVM**	0.85	0.78	0.56	0.67	0.77	0.53	0.69
**Accuracy for PLS**	0.96	0.84	0.68	0.63	0.67	0.63	0.74
**Accuracy for PCA**	0.92	0.82	0.74	0.66	0.75	0.48	0.73
**Accuracy for SVM**	0.88	0.84	0.68	0.63	0.75	0.59	0.73
**F score for PLS**	0.96	0.86	0.70	0.71	0.67	0.62	0.75
**F score for PCA**	0.92	0.83	0.71	0.58	0.79	0.59	0.74
**F score for SVM**	0.87	0.86	0.73	0.63	0.73	0.59	0.74
**AUC for PLS**	0.972	0.856	0.767	0.739	0.748	0.628	0.79
**AUC for PCA**	0.951	0.881	0.792	0.675	0.790	0.456	0.76
**AUC for SVM**	0.944	0.850	0.706	0.665	0.755	0.528	0.74
**AUC Average for PCA, SVM, and PLS**	**0.956**	**0.862**	**0.755**	**0.693**	**0.765**	**0.537**	**0.76**

**Table 4:** Comparison of SERS-based classification performance with that in previously reported studies.

Reference	SERS Application	Substrate	ML/ Chemometrics	AUC	Accuracy	EF	*N* Samples Measured
Present publication: Boranova et al.	Albuminuria/urine	D200 AuNPs on Au	PLS-LDA	0.97	0.96	5.4 × 10^7^	77
		λ650 AuNRs on Au	PLS-LDA	0.86	0.84	11 × 10^6^	124
		λ750 AuNRs on Au	PCA-LDA	0.79	0.74	3 × 10^6^	124
		Plus results on 3 other substrates with lower performance and a total of 289 samples.					
Zong et al. [25]	Chronic kidney disease/urine	35 ± 5 nm AgNPs on aluminum	PCA-LDA	0.89	0.82	NA	187
Moisoiu et al. [21]	Breast cancer/urine	Ag NPs on aluminum	PCA-LDA	NA	0.88	NA	75
Qiu et al. [88]	Kidney stones/urine	AgNPs on aluminum	PCA-SVM	0.95	0.92	NA	98
Ma et al. [89]	Prostate cancer/urine	AgNPs on silicon	GA-PLS-LDA	0.96	0.9	NA	75
Zhang et al. [43]	Bladder cancer/serum	AgNPs on aluminum	SVM	0.95	0.95	NA	88
Yan et al. [39]	Neuropsychiatric disorders/serum	Au@AgNAs on aluminum	PLS-LDA	0.97	0.92	NA	119

NAs are nanoaggregates; ML is Machine Learning.

## Data Availability

Data will be made available on request.

## Supplementary Materials

The following supporting information can be downloaded at https://www.mdpi.com/article/10.3390/ijms27198833/s1. References [85,87] are cited in the Supplementary Materials.
