# Supplementary material for: Comparison of SERS Substrates in Direct Analysis of Urine Samples for Detection of Albuminuria: Relatively Large Gold Nano-Spheroids Outperform Gold Nanorods on Gold Film
Source: Int J Mol Sci. 2026 Oct 3;27(19):8833. doi: 10.3390/ijms27198833 (PMC13665429; doi:10.3390/ijms27198833)
Supplement: Supplementary file 1 [file ijms-27-08833-s001.zip › ijms-4505039-supplementary.pdf]

Supplementary Information for

Comparison of SERS Substrates in Direct Analysis of Urine Samples, Applied for Detection of Albuminuria : Relatively Large Gold Nano-Spheroids Outperform Gold Nanorods on Gold Film

Aigerim Boranova[1], Almat Kabyl[1], Bagdat Tuleuov[1], Madiyar Kassymaly[1], Zhalaliddin Makhammajanov[2], Abduzhappar Gaipov[2] and Rostislav Bukasov[1] *

[1]Department of Chemistry, School of Sciences and Humanities (SSH) Nazarbayev University, Astana, Kazakhstan; [2]Department of Medicine, School of Medicine, Nazarbayev University, Astana, Kazakhstan.

* Corresponding: rostislav.bukasov@nu.edu.kz

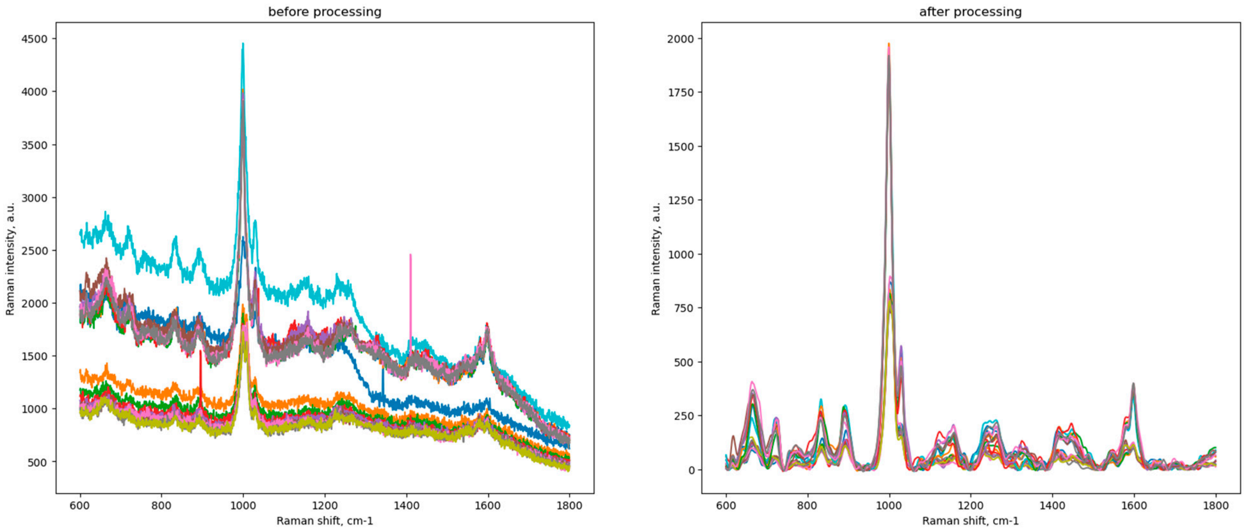

Figure S1. Spectroscopic data processing

Figure 1 shows spectroscopic data processing where an asymmetric least squares algorithm was used to exclude fluorescent background, whereas a median filter was implemented to remove outliers and the Savitsky-Golay smoothing algorithm was applied to smooth the desired signal.

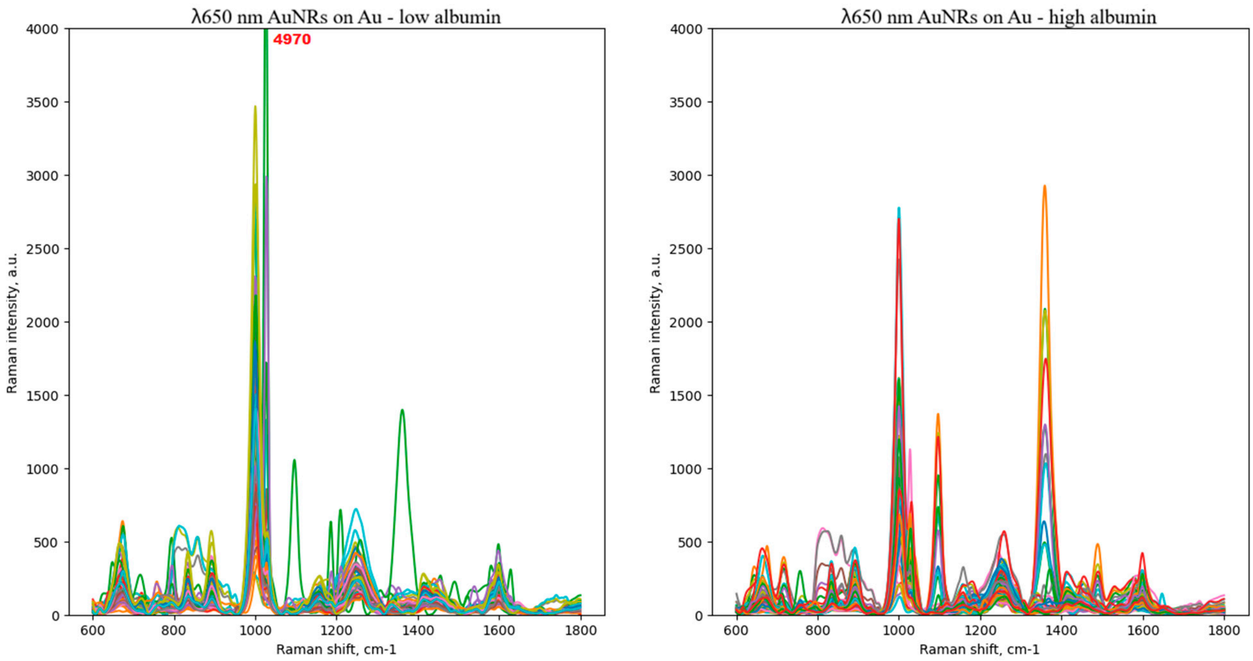

Figure S2. Spectra high vs low albumin groups of λ650 nm AuNRs on Au

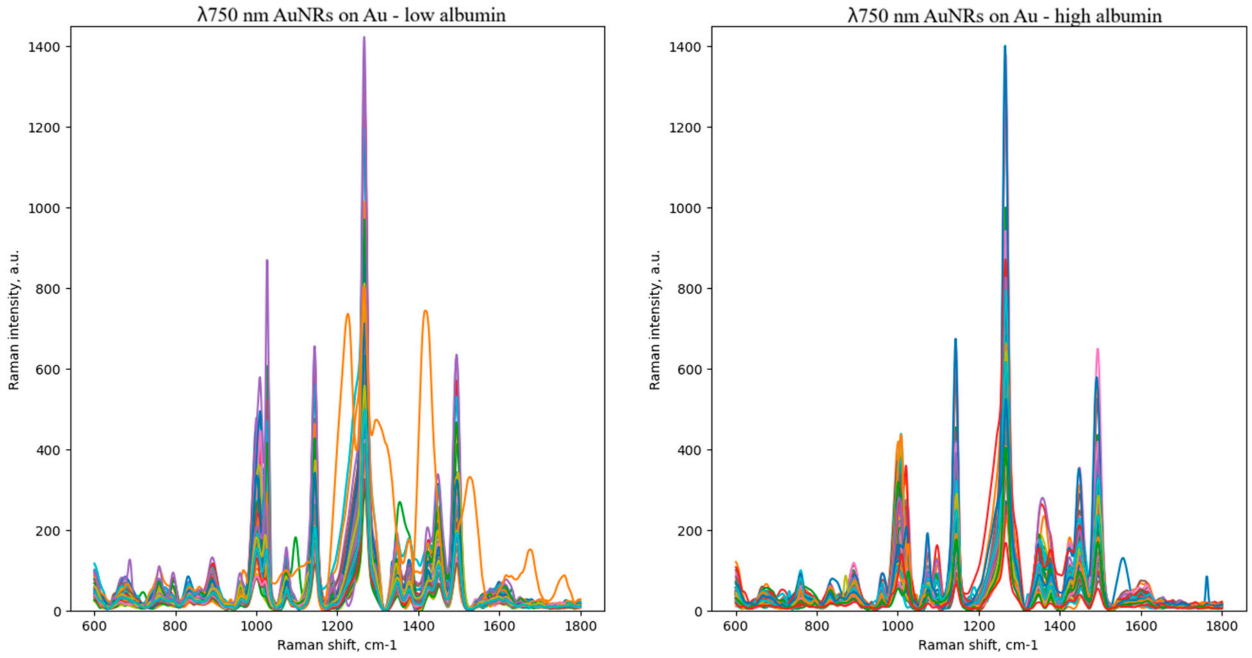

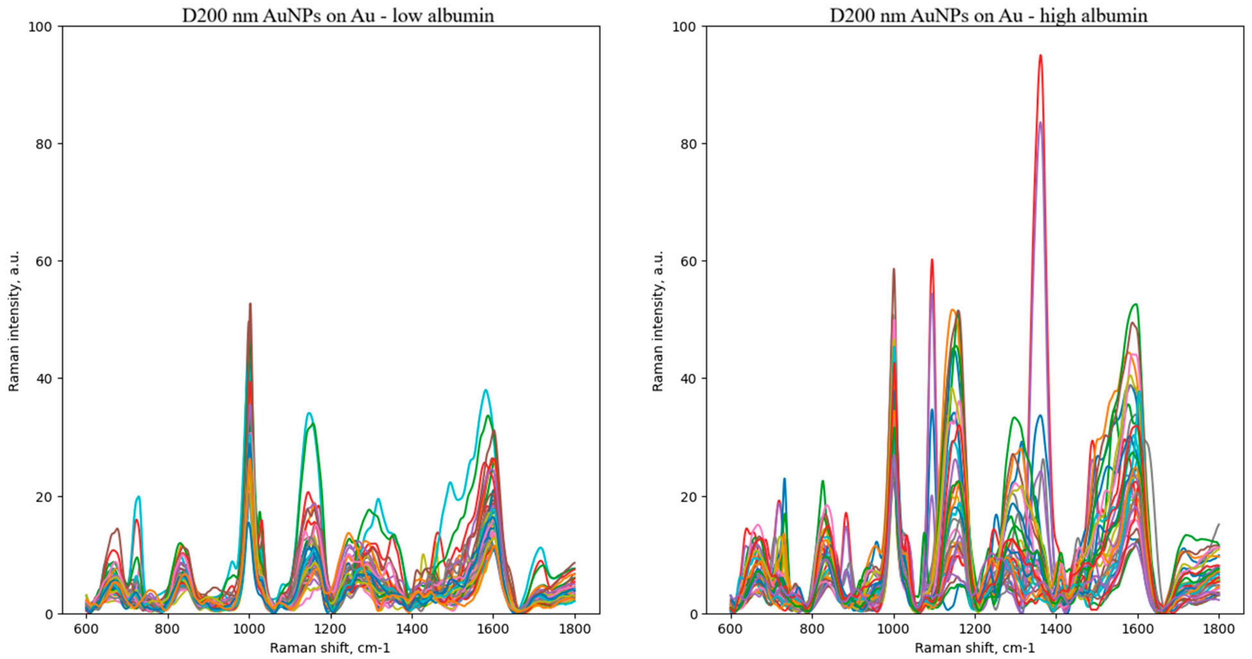

Figure S3. Spectra high vs low albumin groups of λ750 nm AuNRs on Au, and D200 nm AuNPs on Au

The higher signals of the nanorods can be explained by the double and triple stacking of the nanorods on the surface, which creates a large number of small nanogaps that are likely to create a significant number of strong hot spots.

Table S1 to Table S5 bellow all use 10-fold cross validation to calculate the results

Table S1. A summarized table of classification performance for the set λ650 nm Au NRs on Au substrate for (A) PLS-LDA model, (B) PCA-LDA model and (C) PLS-SVM model

A	PLS-LDA Results for λ650 AuNRs on Au				N samples=38				
Component	Sensitivity	Specificity	Accuracy	AUC	TP	TN	FP	FN	LOOCV MSE
1	0.850	0.722	0.789	0.831	17	13	5	3	0.2214
2	0.900	0.667	0.789	0.828	18	12	6	2	0.2066
3	0.900	0.778	0.842	0.856	18	14	4	2	0.1820
4	0.850	0.722	0.789	0.856	17	13	5	3	0.1759
5	0.750	0.833	0.789	0.814	15	15	3	5	0.1756
6	0.800	0.611	0.711	0.811	16	11	7	4	0.1884
7	0.650	0.778	0.711	0.828	13	14	4	7	0.1870
8	0.850	0.667	0.763	0.831	17	12	6	3	0.1910
9	0.800	0.611	0.711	0.844	16	11	7	4	0.1959
10	0.800	0.611	0.711	0.836	16	11	7	4	0.1838

B	PCA-LDA Results for λ650 AuNRs on Au				N samples =38				
Component	Sensitivity	Specificity	Accuracy	AUC	TP	TN	FP	FN	LOOCV MSE
1	0.650	0.611	0.632	0.678	13	11	7	7	0.2389
2	0.900	0.611	0.763	0.806	18	11	7	2	0.2256
3	0.900	0.500	0.711	0.825	18	9	9	2	0.2233

4	0.800	0.722	0.763	0.817	16	13	5	4	0.2308
5	0.950	0.667	0.816	0.842	19	12	6	1	0.2197
6	0.850	0.778	0.816	0.881	17	14	4	3	0.2087
7	0.800	0.833	0.816	0.883	16	15	3	4	0.2205
8	0.850	0.722	0.789	0.828	17	13	5	3	0.1828
9	0.950	0.333	0.658	0.831	19	6	12	1	0.1778
10	0.750	0.833	0.789	0.825	15	15	3	5	0.1772

C	PLS-SVM Results for λ650 AuNRs on Au				N samples=38				
Component	Sensitivity	Specificity	Accuracy	AUC	TP	TN	FP	FN	LOOCV MSE
1	0.850	0.722	0.789	0.831	17	13	5	3	0.2207
2	0.900	0.611	0.763	0.817	18	11	7	2	0.2072
3	0.900	0.722	0.816	0.856	18	13	5	2	0.1773
4	0.900	0.778	0.842	0.850	18	14	4	2	0.1800
5	0.900	0.500	0.711	0.804	18	9	9	2	0.1743
6	0.900	0.556	0.737	0.779	18	10	8	2	0.1705
7	0.900	0.444	0.684	0.781	18	8	10	2	0.1660
8	0.900	0.611	0.763	0.808	18	11	7	2	0.1719
9	0.850	0.611	0.737	0.844	17	11	7	3	0.1753
10	0.850	0.611	0.737	0.828	17	11	7	3	0.1760

Table S2. A summarized table of classification performance for the set λ650 nm Au NRs on Ag substrate for (A) PLS-LDA model, (B) PCA-LDA model and (C) PLS-SVM model

A	PLS Results for λ650 AuNRs on Ag				N samples=38				
Component	Sensitivity	Specificity	Accuracy	AUC	TP	TN	FP	FN	LOOCV MSE
1	0.400	0.778	0.579	0.622	8	14	4	12	0.2471
2	0.850	0.111	0.500	0.689	17	2	16	3	0.2481
3	0.850	0.222	0.553	0.672	17	4	14	3	0.2484
4	0.850	0.389	0.632	0.739	17	7	11	3	0.2448
5	0.850	0.333	0.605	0.667	17	6	12	3	0.2703
6	0.800	0.333	0.579	0.597	16	6	12	4	0.2634
7	0.700	0.500	0.605	0.653	14	9	9	6	0.2530
8	0.300	0.944	0.605	0.642	6	17	1	14	0.2670
9	0.300	0.944	0.605	0.561	6	17	1	14	0.2886
10	0.300	0.944	0.605	0.553	6	17	1	14	0.2987

B	PCA Results for λ650 AuNRs on Ag				N samples=38				
Component	Sensitivity	Specificity	Accuracy	AUC	TP	TN	FP	FN	LOOCV MSE
1	0.400	0.722	0.553	0.608	8	13	5	12	0.2475
2	0.450	0.889	0.658	0.675	9	16	2	11	0.2472
3	0.300	0.889	0.579	0.664	6	16	2	14	0.2543
4	0.800	0.222	0.526	0.558	16	4	14	4	0.2434
5	0.750	0.333	0.553	0.564	15	6	12	5	0.2458
6	0.850	0.333	0.605	0.714	17	6	12	3	0.2459
7	0.400	0.944	0.658	0.697	8	17	1	12	0.2582

C	PLS-SVM Results for λ650 AuNRs on Ag				N samples=38				
Component	Sensitivity	Specificity	Accuracy	AUC	TP	TN	FP	FN	LOOCV MSE
1	0.000	1.000	0.474	0.378	0	18	0	20	0.2672
2	0.850	0.111	0.500	0.644	17	2	16	3	0.2474
3	0.050	0.944	0.474	0.647	1	17	1	19	0.2472
4	0.900	0.278	0.605	0.718	18	5	13	2	0.2436
5	0.900	0.167	0.553	0.688	18	3	15	2	0.2513
6	0.600	0.667	0.632	0.665	12	12	6	8	0.2540
7	0.900	0.167	0.553	0.706	18	3	15	2	0.2485
8	0.250	0.944	0.579	0.619	5	17	1	15	0.2594
9	0.300	0.944	0.605	0.650	6	17	1	14	0.2568
10	0.250	0.944	0.579	0.603	5	17	1	15	0.2564

Table S3. A summarized table of classification performance for the set λ750 nm Au NRs on Au substrate for (A) PLS-LDA model, (B) PCA-LDA model and (C) PLS-SVM model

A	PLS Results for λ750 AuNRs on Au				N samples=38				
Component	Sensitivity	Specificity	Accuracy	AUC	TP	TN	FP	FN	LOOCV MSE
1	0.750	0.444	0.605	0.614	15	8	10	5	0.2459
2	0.400	0.833	0.605	0.717	8	15	3	12	0.2358
3	0.450	0.889	0.658	0.722	9	16	2	11	0.2237
4	0.550	0.722	0.632	0.708	11	13	5	9	0.2347
5	0.700	0.667	0.684	0.767	14	12	6	6	0.2223
6	0.550	0.778	0.658	0.717	11	14	4	9	0.2081
7	0.800	0.389	0.605	0.658	16	7	11	4	0.2610
8	0.750	0.333	0.553	0.642	15	6	12	5	0.2721
9	0.800	0.278	0.553	0.614	16	5	13	4	0.2912
10	0.700	0.278	0.500	0.586	14	5	13	6	0.3068

B	PCA Results for λ750 AuNRs on Au				N samples =38				
Component	Sensitivity	Specificity	Accuracy	AUC	TP	TN	FP	FN	LOOCV MSE
1	0.950	0.278	0.632	0.608	19	5	13	1	0.2489
2	0.350	0.778	0.553	0.619	7	14	4	13	0.2517
3	0.900	0.222	0.579	0.594	18	4	14	2	0.2551
4	0.400	0.833	0.605	0.714	8	15	3	12	0.2391
5	0.500	0.833	0.658	0.658	10	15	3	10	0.2452
6	0.450	0.889	0.658	0.703	9	16	2	11	0.2435
7	0.450	0.889	0.658	0.714	9	16	2	11	0.2394
8	0.450	0.889	0.658	0.692	9	16	2	11	0.2257
9	0.500	0.889	0.684	0.775	10	16	2	10	0.2302
10	0.500	0.889	0.684	0.764	10	16	2	10	0.2223
11	0.450	0.889	0.658	0.769	9	16	2	11	0.2216
12	0.500	0.833	0.658	0.775	10	15	3	10	0.2161
13	0.500	0.889	0.684	0.792	10	16	2	10	0.2161
14	0.600	0.889	0.737	0.792	12	16	2	8	0.2197

15	0.550	0.889	0.711	0.792	11	16	2	9	0.2232

C	PLS-SVM Results for λ750 AuNRs on Au				N samples=38				
Component	Sensitivity	Specificity	Accuracy	AUC	TP	TN	FP	FN	LOOCV MSE
1	0.800	0.333	0.579	0.613	16	6	12	4	0.2539
2	0.400	0.944	0.658	0.719	8	17	1	12	0.2329
3	0.800	0.556	0.684	0.706	16	10	8	4	0.2385
4	0.500	0.667	0.579	0.700	10	12	6	10	0.2227
5	0.550	0.611	0.579	0.694	11	11	7	9	0.2346
6	0.750	0.500	0.632	0.700	15	9	9	5	0.1987
7	0.800	0.444	0.632	0.672	16	8	10	4	0.2199
8	0.750	0.556	0.658	0.661	15	10	8	5	0.2188
9	0.800	0.278	0.553	0.608	16	5	13	4	0.2586
10	0.800	0.278	0.553	0.628	16	5	13	4	0.2356

Table S4. A summarized table of classification performance for the set λ650 nm Au NPs on Si substrate for (A) PLS-LDA model, (B) PCA-LDA model and (C) PLS-SVM model

A	PLS Results for λ650 AuNRs on Si				N samples=27				
Component	Sensitivity	Specificity	Accuracy	AUC	TP	TN	FP	FN	LOOCV MSE
1	0.833	0.200	0.481	0.739	10	3	12	2	0.2597
2	0.833	0.200	0.481	0.739	10	3	12	2	0.2403
3	0.833	0.333	0.556	0.650	10	5	10	2	0.2355
4	0.667	0.600	0.630	0.628	8	9	6	4	0.2178
5	0.500	0.667	0.593	0.633	6	10	5	6	0.2166
6	0.667	0.533	0.593	0.517	8	8	7	4	0.2423
7	0.667	0.467	0.556	0.461	8	7	8	4	0.2630
8	0.500	0.533	0.519	0.539	6	8	7	6	0.2713
9	0.500	0.533	0.519	0.561	6	8	7	6	0.2711
10	0.500	0.733	0.630	0.639	6	11	4	6	0.2320

B	PCA Results for λ650 AuNRs on Si				N samples=27				
Component	Sensitivity	Specificity	Accuracy	AUC	TP	TN	FP	FN	LOOCV MSE
1	0.000	1.000	0.556	0.256	0	15	0	12	0.2547
2	0.000	1.000	0.556	0.750	0	15	0	12	0.2410
3	0.833	0.200	0.481	0.456	10	3	12	2	0.2451

C	PLS-SVM Results for λ650 AuNRs on Si				N samples=27				
Component	Sensitivity	Specificity	Accuracy	AUC	TP	TN	FP	FN	LOOCV MSE
1	0.000	1.000	0.556	0.739	0	15	0	12	0.2475
2	0.833	0.200	0.481	0.739	10	3	12	2	0.2496
3	0.583	0.667	0.630	0.611	7	10	5	5	0.2342
4	0.000	1.000	0.556	0.622	0	15	0	12	0.2372
5	0.417	0.667	0.556	0.567	5	10	5	7	0.2360
6	0.667	0.533	0.593	0.528	8	8	7	4	0.2173

7	0.583	0.533	0.556	0.500	7	8	7	5	0.2417
8	0.833	0.200	0.481	0.689	10	3	12	2	0.2410
9	0.917	0.133	0.481	0.594	11	2	13	1	0.2357
10	0.000	0.933	0.519	0.578	0	14	1	12	0.2416

Table S5. A summarized table of classification performance for the set D200 nm Au NPs on Al substrate for (A) PLS-LDA model, (B) PCA-LDA model and (C) PLS-SVM model

A	PLS Results for D200 AuNPs on Al				N samples=24				
Component	Sensitivity	Specificity	Accuracy	AUC	TP	TN	FP	FN	LOOCV MSE
1	0.545	0.769	0.667	0.755	6	10	3	5	0.2498
2	0.727	0.615	0.667	0.748	8	8	5	3	0.2633
3	0.545	0.538	0.542	0.573	6	7	6	5	0.3480
4	0.727	0.308	0.500	0.580	8	4	9	3	0.3689
5	0.636	0.538	0.583	0.573	7	7	6	4	0.3600
6	0.727	0.231	0.458	0.545	8	3	10	3	0.3341
7	0.545	0.308	0.417	0.545	6	4	9	5	0.3513
8	0.636	0.231	0.417	0.545	7	3	10	4	0.3624
9	0.545	0.692	0.625	0.580	6	9	4	5	0.3779
10	0.545	0.769	0.667	0.573	6	10	3	5	0.4161

B	PCA Results for D200 AuNPs on Al				N samples=24				
Component	Sensitivity	Specificity	Accuracy	AUC	TP	TN	FP	FN	LOOCV MSE
1	0.727	0.538	0.625	0.748	8	7	6	3	0.2466
2	0.909	0.538	0.708	0.783	10	7	6	1	0.2491
3	1.000	0.538	0.750	0.790	11	7	6	0	0.2495
4	0.818	0.462	0.625	0.727	9	6	7	2	0.2531
5	0.909	0.462	0.667	0.797	10	6	7	1	0.2563
6	0.909	0.462	0.667	0.811	10	6	7	1	0.2610
7	1.000	0.385	0.667	0.790	11	5	8	0	0.2771
8	0.818	0.462	0.625	0.783	9	6	7	2	0.2873
9	1.000	0.385	0.667	0.790	11	5	8	0	0.2893
10	0.818	0.769	0.792	0.818	9	10	3	2	0.3012

C	PLS-SVM Results for D200 AuNPs on Al				N samples=24				
Component	Sensitivity	Specificity	Accuracy	AUC	TP	TN	FP	FN	LOOCV MSE
1	0.727	0.769	0.750	0.755	8	10	3	3	0.2466
2	1.000	0.385	0.667	0.748	11	5	8	0	0.2548
3	0.545	0.462	0.500	0.615	6	6	7	5	0.3078
4	0.636	0.385	0.500	0.601	7	5	8	4	0.3089
5	0.727	0.308	0.500	0.601	8	4	9	3	0.3105
6	0.727	0.308	0.500	0.601	8	4	9	3	0.2936
7	0.727	0.308	0.500	0.559	8	4	9	3	0.2984
8	0.545	0.462	0.500	0.650	6	6	7	5	0.3086
9	0.636	0.231	0.417	0.615	7	3	10	4	0.3208
10	0.545	0.308	0.417	0.601	6	4	9	5	0.3278

Table S6. A summarized table of classification performance for all substrates for (A) Leave-One-Out Cross-Validation, (B) 5-fold cross-validation

A	Leave-One-Out Cross-Validation						
	**D200 nm AuNPs on Au**	**λ650 nm AuNRs on Au**	**λ750 nm AuNRs on Au**	**λ650 nm AuNRs on Ag**	**D200 nm AuNPs on Al**	**λ650 nm AuNRs on Si**	**Average**
**Number of samples**	77	124	124	124	77	88	
**Sensitivity PLS***	1.00	0.85	0.75	0.65	1.00	0.67	0.82
**Sensitivity PCA***	1.00	0.85	0.70	0.40	1.00	0.83	0.80
**Sensitivity SVM***	0.91	0.90	0.70	0.40	0.73	0.75	0.73
**Specificity PLS***	0.92	0.78	0.61	0.78	0.38	0.53	0.67
**Specificity PCA ***	0.85	0.78	0.67	0.94	0.62	0.20	0.68
**Specificity SVM***	0.85	0.78	0.56	0.94	0.62	0.47	0.70
**Accuracy PLS ***	0.96	0.82	0.68	0.71	0.67	0.59	0.74
**Accuracy PCA ***	0.92	0.82	0.68	0.66	0.79	0.48	0.72
**Accuracy SVM ***	0.88	0.84	0.63	0.66	0.67	0.59	0.71
**Accuracy Average PLS and SVM**	0.92	0.83	0.66	0.68	0.67	0.59	
**F score PLS**	0.96	0.83	0.71	0.70	0.73	0.59	0.75
**F score PCA**	0.92	0.83	0.70	0.55	0.81	0.59	0.73
**F score SVM**	0.87	0.86	0.67	0.55	0.67	0.62	0.71
**AUC PLS ***	0.972	0.856	0.767	0.739	0.748	0.517	0.766
**AUC PCA ***	0.965	0.883	0.764	0.714	0.790	0.750	0.811
**AUC SVM ***	0.944	0.850	0.661	0.650	0.748	0.500	0.726
**AUC average PLS and SVM**	**0.958**	**0.853**	**0.714**	**0.694**	**0.748**	**0.508**	0.746
**AUC Average PCA and PLS**	**0.969**	**0.869**	**0.765**	**0.726**	**0.769**	**0.633**	0.789
**AUC Average PCA, SVM and PLS**	**0.960**	**0.863**	**0.731**	**0.701**	**0.762**	**0.589**	0.768
*Calculated for PC number which provides minimum MSE							

B	5-fold cross-validation						
	**D200 nm AuNPs on Au**	**λ650 nm AuNRs on Au**	**λ750 nm AuNRs on Au**	**λ650 nm AuNRs on Ag**	**D200 nm AuNPs on Al**	**λ650 nm AuNRs on Si**	**Average**
**Number of samples**	77	124	124	124	77	88	
**Sensitivity PLS***	0.91	0.90	0.70	0.60	1.00	0.67	0.80
**Sensitivity PCA***	1.00	0.85	0.60	0.85	0.91	0.83	0.84
**Sensitivity SVM***	0.55	0.90	0.75	0.60	0.91	0.58	0.71
**Specificity PLS***	0.85	0.78	0.72	0.78	0.38	0.67	0.70
**Specificity PCA ***	0.85	0.78	0.89	0.33	0.54	0.20	0.60
**Specificity SVM***	1.00	0.72	0.39	0.67	0.54	0.67	0.66
**Accuracy PLS ***	0.88	0.84	0.71	0.68	0.67	0.67	0.74
**Accuracy PCA ***	0.92	0.82	0.74	0.61	0.71	0.48	0.71
**Accuracy SVM ***	0.79	0.82	0.58	0.63	0.71	0.63	0.69
**Accuracy Average PLS and SVM**	0.83	0.83	0.64	0.66	0.69	0.65	
**F score PLS**	0.87	0.86	0.72	0.67	0.73	0.64	0.75
**F score PCA**	0.92	0.83	0.71	0.69	0.74	0.59	0.75
**F score SVM**	0.71	0.84	0.65	0.63	0.74	0.58	0.69
**AUC PLS ***	0.944	0.856	0.767	0.739	0.748	0.633	0.781
**AUC PCA ***	0.965	0.883	0.792	0.714	0.783	0.456	0.765
**AUC SVM ***	0.944	0.850	0.700	0.665	0.748	0.567	0.746
**AUC average PLS and SVM**	**0.944**	**0.853**	**0.733**	**0.702**	**0.748**	**0.600**	0.763
**AUC Average PCA and PLS**	**0.955**	**0.869**	**0.779**	**0.726**	**0.766**	**0.544**	0.773
**AUC Average PCA, SVM and PLS**	**0.951**	**0.863**	**0.753**	**0.706**	**0.760**	**0.552**	**0.764**
*Calculated for PC number which provides minimum MSE							

Table S7 Correlation of AUC Average (PCA, SVM and PLS) vs EFs calculated for NBT for Three various nanoparticle/nanorods on gold film; same as the Three top performing substrates.

	D200 nm	λ650 NR	λ750 NR	
EF x10^6	54.2	11.2	2.71	Correlation
AUC with 10-fold	0.956	0.862	0.755	**0.918**
AUC with 5-fold	0.96	0.863	0.731	**0.897**
AUC with Leave Out	0.951	0.863	0.753	**0.908**
Average AUC	0.956	0.863	0.746	**0.908**
stdev	0.0045	0.0006	0.0133	0.0106
Relative stdev, %	0.5%	0.1%	1.8%	1.2%

"Conclusion: 10-fold cross validation achieves the best correlation of AUC vs EF,(0.918) However, variability in values of AUC is relatively small (within 0.5 % difference from the mean) with only one exception (λ750 NR, 5-fold, difference from the mean 1.5%). The relative standard deviations of mean AUCs calculated for each kind of substrate is only above 0.5% for λ750 NRs (1.8%) because of this exception , which demonstrates good similarity in classification performance of 3 different cross-validation method obtained for 3 top performing SERS substrates."

.

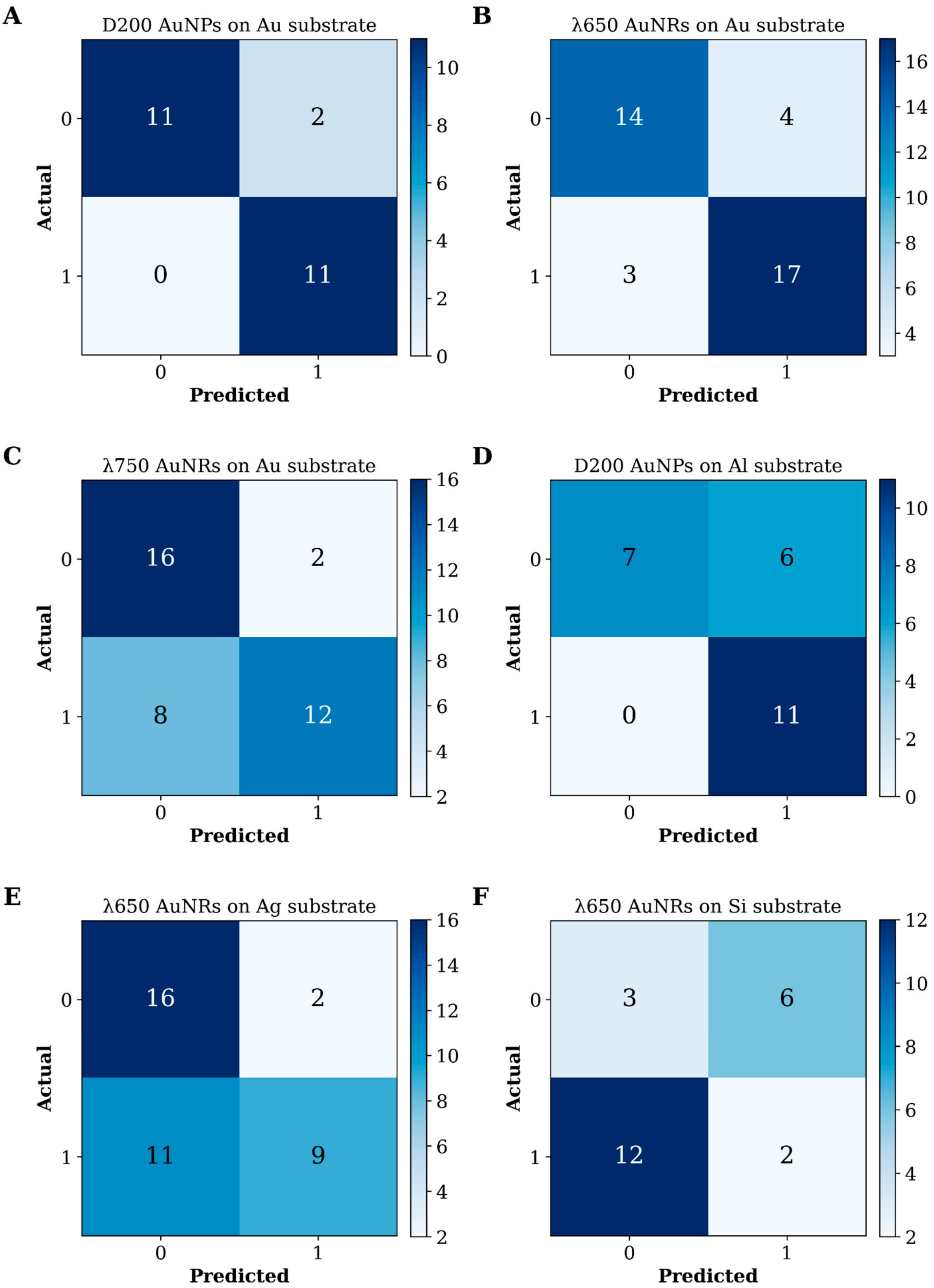

Figure S4. Confusion matrices of PCA-LDA classification results. (A) D200 AuNPs on Au substrate (B) λ650 AuNRs on Au substrate (C) λ750 AuNRs on Au substrate (D) D200 AuNPs on Al substrate (E) λ650 AuNRs on Ag substrate (F) λ650 AuNRs on Si substrate.

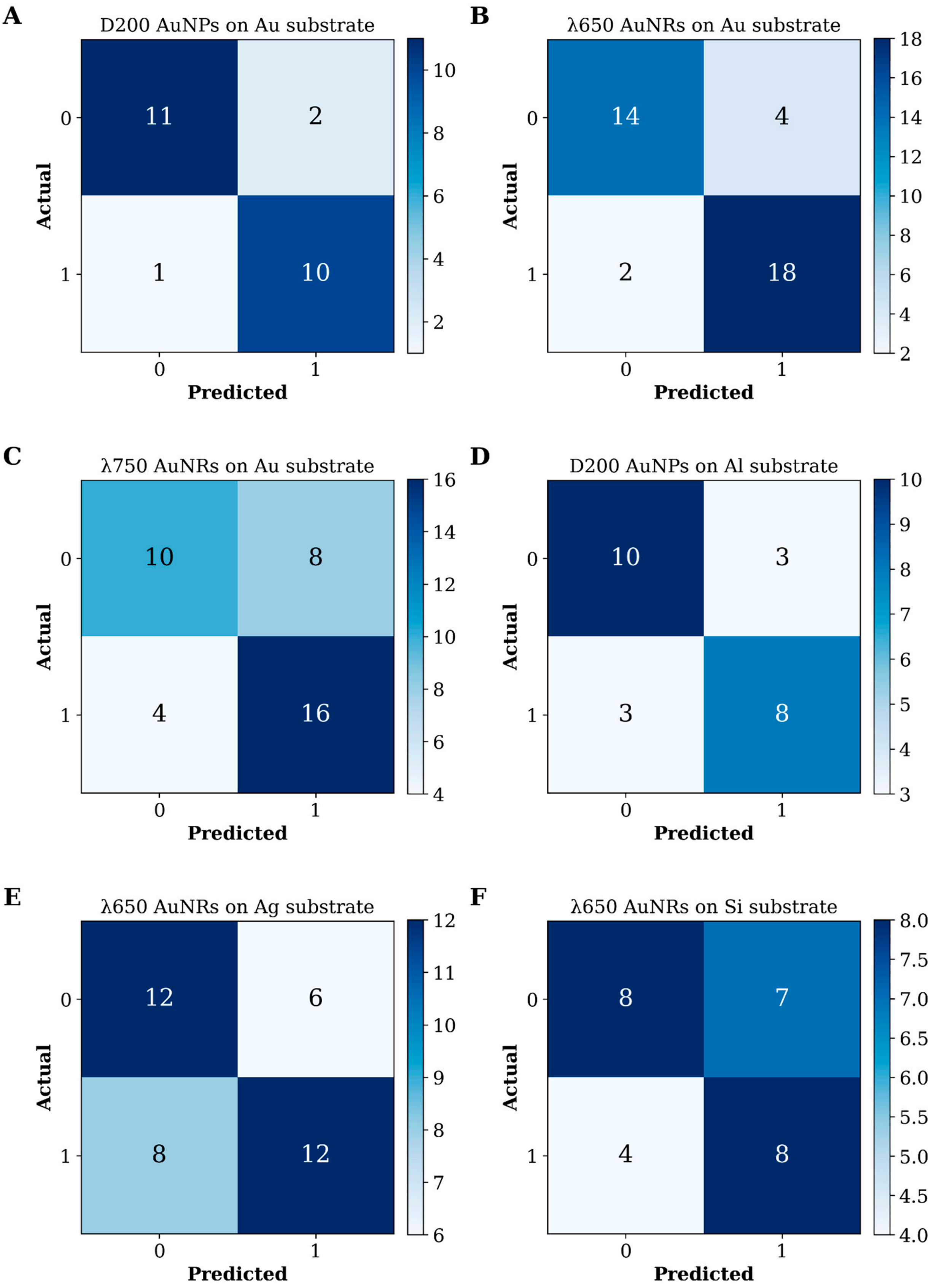

Figure S5. Confusion matrices of PLS-SVM classification results. (A) D200 AuNPs on Au substrate (B) λ650 AuNRs on Au substrate (C) λ750 AuNRs on Au substrate (D) D200 AuNPs on Al substrate (E) λ650 AuNRs on Ag substrate (F) λ650 AuNRs on Si substrate.

Enhancement Factor (EF) Calculation

Fundamentally, enhancement factor (EF) is calculated as follows [1,2]:

$$EF = \frac{I_{SERS}}{I_{Bulk}} \cdot \frac{N_{Bulk}}{N_{SERS}}$$

where I_{SERS} — background-adjusted Raman intensity of the maximum peak of an analyte measured on a SERS substrate, I_{Bulk} — background-adjusted unenhanced Raman intensity of the maximum peak of an analyte, N_{SERS} — number of analyte molecules that contribute to the SERS signal, N_{Bulk} — number of analyte molecules that contribute to the unenhanced Raman signal[1].

Let A be the area of the laser spot. Then, the number N_{Bulk} is calculated as

$$N_{Bulk} = \frac{Ah\rho N_A}{M} = AhNv_{Bulk}$$

where h — confocal depth, ρ — density of an analyte, M — molar mass of an analyte, N_A — Avogadro number, Nv_{Bulk}— number of analyte molecules in a unit volume.

Let r be the radius of a liquid droplet on the gold substrate after drying. Assume that the analyte molecules are equally distributed in that droplet. Then, the number N_{SERS} is calculated as

$$N_{SERS} = \frac{cVN_AA}{\pi r^2} = C_sA$$

where c— concentration of analyte molecules in the liquid droplet, V — volume of the droplet, C_s— number of analyte molecules in a unit area.

We get the final expression for enhancement factor calculation by incorporating the equations (2) and (3) into the equation (1):

$$EF = \frac{I_{SERS}}{I_{Bulk}} \cdot \frac{Nv_{Bulk}h}{C_s}$$

The results of the EF calculations are given in table 7 below:

Table S8. The results of the EF calculations for 60,100,200 nm AuNPs, and 650λ,750λ nm AuNRs (all on gold film) with 4-NBT as an analyte.

**Concentration of NBT, nM**	**Nanoparticle**	I_{SERS}	I_{Bulk}	Nv_{Bulk}, **molecules/µm3**	C_s, **molecules/µm2**	**EF**	**S/N**
10	200 nm AuNPs	312,07	2459,2	5,28x10^9	4,26x10^3	5,42x10^7	41
10	100 nm AuNPs	16.47	2459.2	5,28x10^9	4,26x10^3	2.86x10^6	17
1000*	60 nm AuNPs	217.95	2459.2	5,28x10^9	4,26x10^5	3.79x10^5	42
10	650λ nm AuNRs	64,52	2459,2	5,28x10^9	4,26x10^3	1,12x10^7	72
10	750λ nm AuNRs	15,58	2459,2	5,28x10^9	4,26x10^3	2,71x10^6	25

All measurements are done with 785 nm laser excitation and x10 objectives

The measurement of 10 and 100 nM solutions of NBT on 60 nm AuNPs/Au film substrate gave unsatisfactory low S/N ratio and therefore we had to take higher concentration of NBT: 1000 nM to obtain sufficiently good signal (S/N =41) for EF calculation.

Table 8 below shows the necessary parameters that are needed to calculate Nv_{Bulk} and C_s:

Table S9. The parameters needed for calculation of Nv_{Bulk} and C_s.

$r_{Droplet}$, mm	$M(NBT)$, g/mol	$\rho(NBT)$, g/cm3	$V_{Droplet}$, L	h, µm
1.5	155.17	1.36	5x10^{-6}	345[2]

The background correction was done by drawing a line between the left and right centroids from the "shoulders" of the characteristic peak of 4-NBT (1336 cm^{-1}) and finding the intensity along this line at the characteristic peak. The figure below demonstrates this visually.

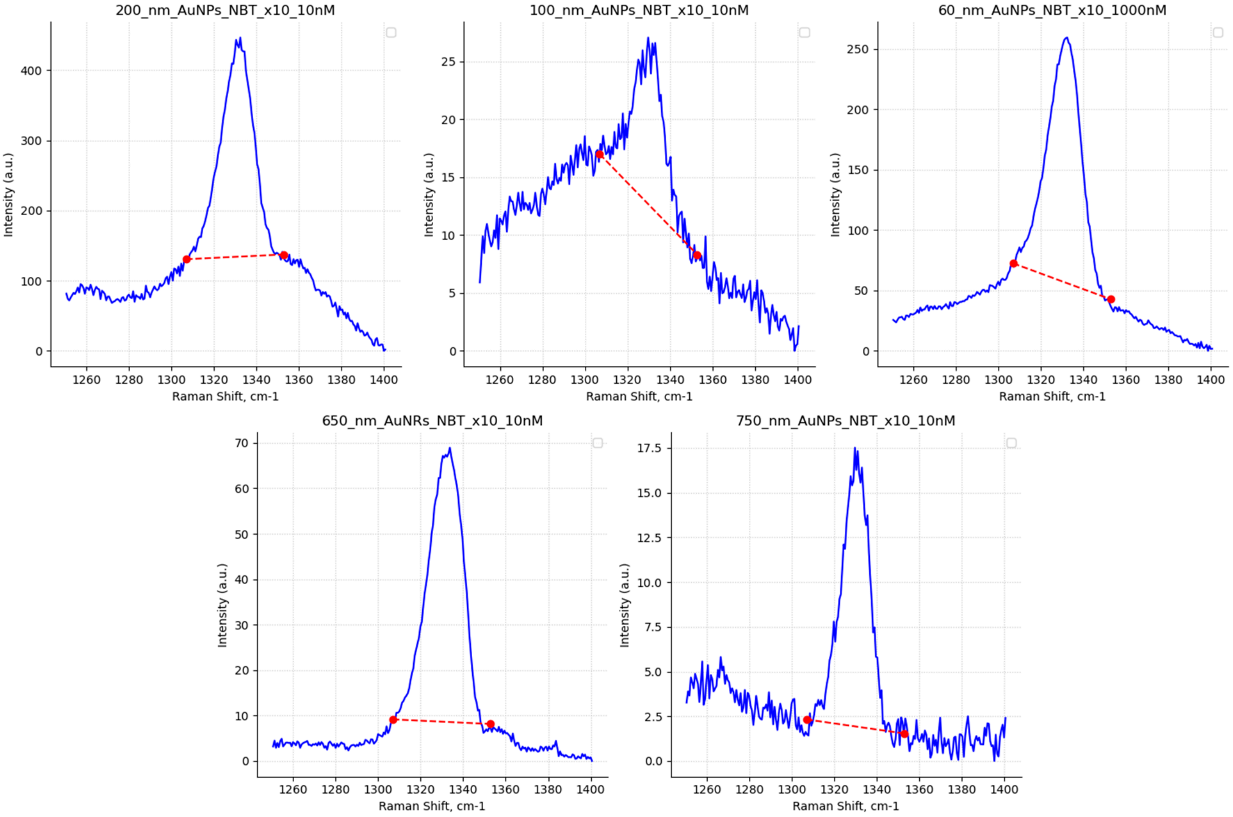

Figure S6. The characteristic averaged Raman (SERS) spectra of 4-NBT on D 200 nm, D 100 nm D 60 nm AuNPs, λ650nm AuNRs, and λ750nm AuNRs on gold film at 10nM concentrations of 4-NBT in all cases, but one (1000 nM for D 60 nm AuNPs).

As an example of the calculation, let's consider the enhancement factor for 650nm AuNRs on a gold substrate with 4-NBT at concentration 10 nM and objective x10. First, the number of bulk analyte molecules in a unit volume is

$$Nv_{Bulk} = \frac{\rho N_A}{M} = \frac{1.36\ \frac{g}{cm^3} \cdot 6.02\ \cdot 10^{23}\ mol^{-1}}{155.17\ \frac{g}{mol}} \cdot 10^{-12}\ \frac{cm^3}{\mu m^3} = 5.28\ \cdot 10^9\ \frac{molecules}{\mu m^3}$$

Second, the number of analyte molecules in a unit area is

$$C_s = \frac{cVN_A}{\pi r^2} = \frac{10^{-8}\ M\ \cdot 5 \cdot 10^{-6}\ L\ \cdot 6.02 \cdot 10^{23}\ mol^{-1}}{\pi \cdot 1.5^2\ mm^2} \cdot 10^{-6}\ \frac{mm^2}{\mu m^2} = 4260\ \frac{molecules}{\mu m^2}$$

Third, the enhancement factor (EF) is

$$EF\ = \frac{64{,}52}{2459{,}15} \cdot \frac{5.28\ \cdot 10^9\ \frac{molecules}{\mu m^3} \cdot 345\ \mu m}{4260\ \frac{molecules}{\mu m^2}} = \ 1.12\ \cdot 10^7$$

Table S10. Statistical analysis of variation in individual Raman spectra for the four types of nanoparticles on gold film

Nanoparticle	Standard Deviation, a.u	Mean, a.u	Relative Standard Deviation (RSD), %	Relative Standard Error, %
200 nm AuNPs	73.45	312.07	23.5	5.9
100 nm AuNPs	6.01	13.65	44.0	11.0
650λ nm AuNRs	24.30	64.52	37.7	9.4
750λ nm AuNRs	3.29	15.58	21.1	5.3

All measurements were performed for aliquots of the same 10 nM solution of NBT drop-casted on each substrate made of specified nanoparticles or nanorods on gold film with objective x10, within one or several days after sample preparation. The Grubbs' test for outliers (95% confidence) revealed that there are no outlier signals. Thus, all 16 Raman spectra (four replicates with four spots) were used for the statistical analysis for each nanoparticle size/morphology.

TEM Images

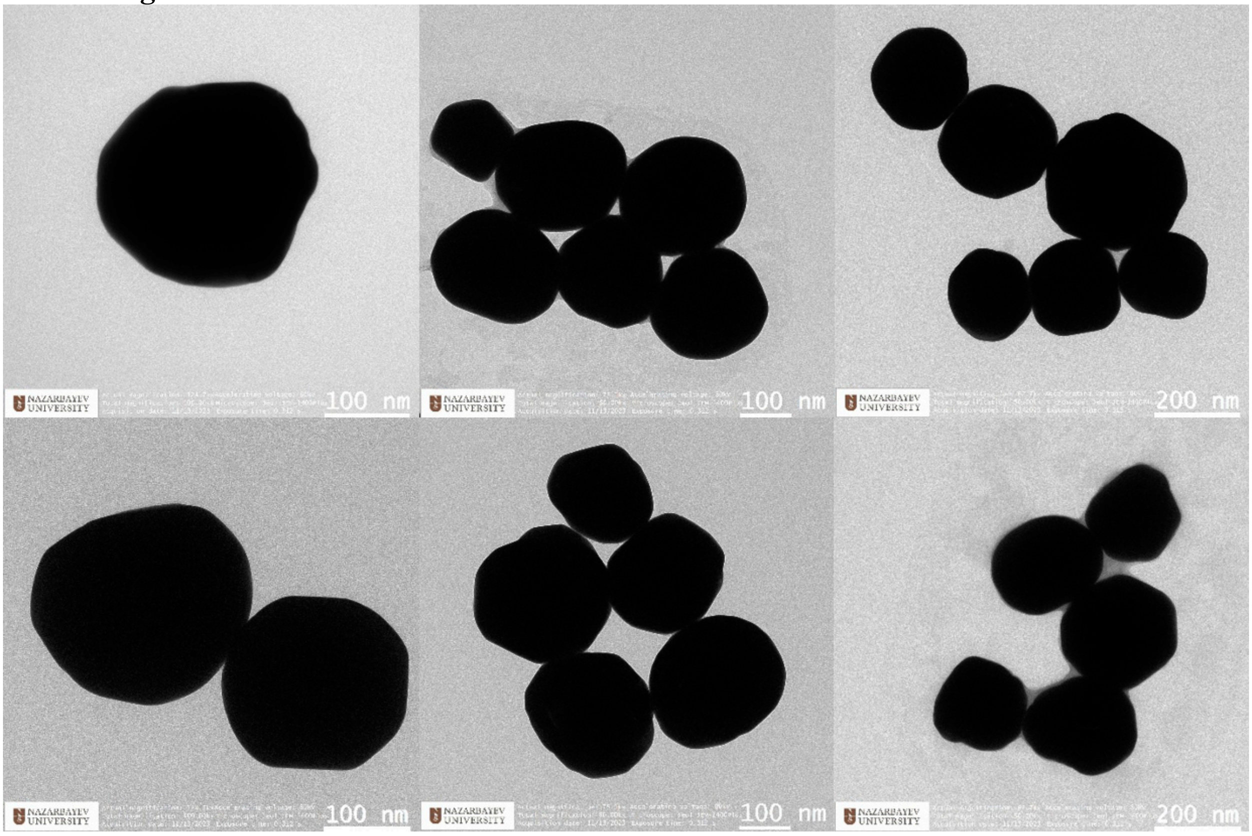

Figure S7. TEM images of D200 nm Au NPs on the gold substrate

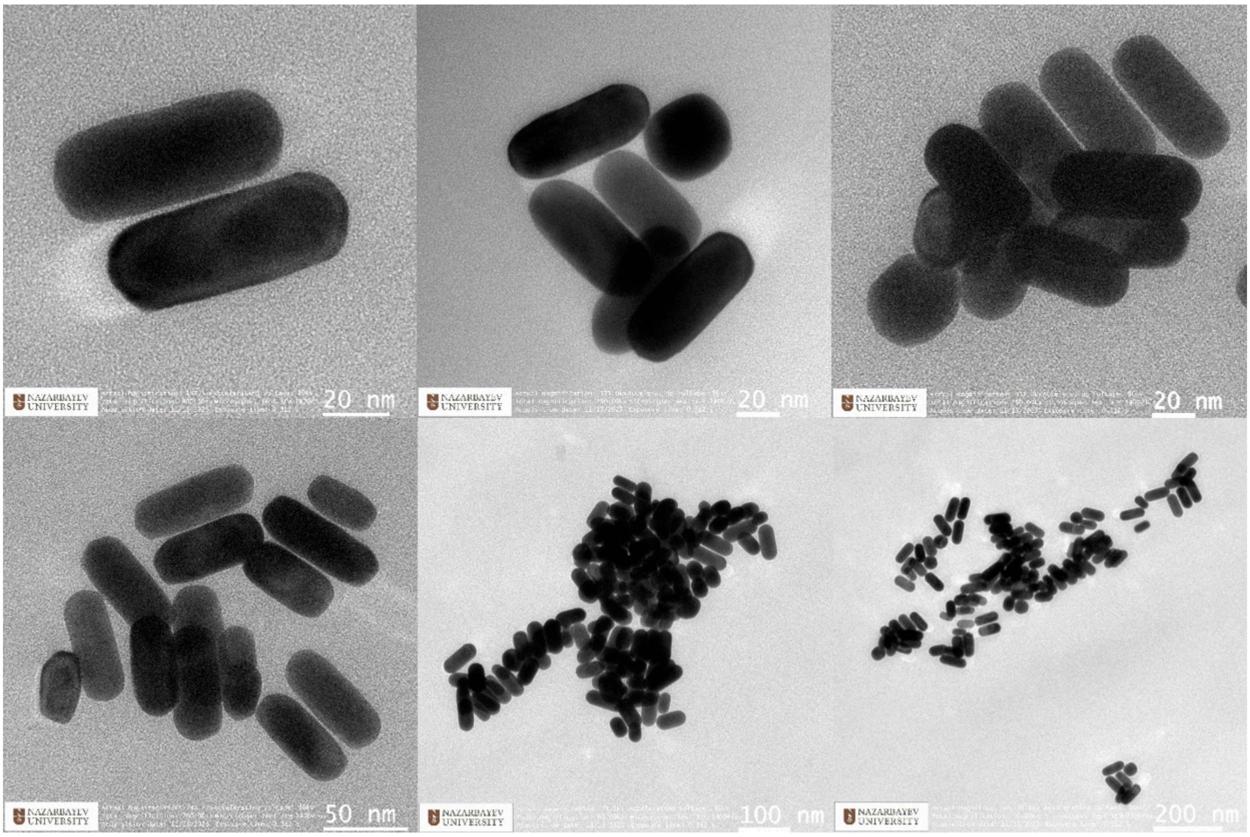

Figure S8. TEM images of λ650 nm Au NRs

According to Figures 7 and 8, the phenomenon of stacking two or three layers of nanoparticles on the surface was sometimes observed only for λ650 nm Au NRs.

REFERENCES

1. Mercedi, A.; Cardoni, F.; Toffanello, F.; Reguera, J.; Meneghetti, M.; Litti, L. Reliable Methodology for Measuring SERS Enhancement Factor on Colloidal and Solid Substrates: A Practical Guide. *Journal of Raman Spectroscopy* **2025**, *56*, 835-849, doi:https://doi.org/10.1002/jrs.6776.
2. Gudun, K.; Elemessova, Z.; Khamkhash, L.; Ralchenko, E.; Bukasov, R. Commercial Gold Nanoparticles on Untreated Aluminum Foil: Versatile, Sensitive, and Cost-Effective SERS Substrate. *Journal of Nanomaterials* **2017**, *2017*, 9182025, doi:10.1155/2017/9182025.
